# Systematic review and meta-analysis of Tuberculosis and COVID-19 Co-infection: Prevalence, fatality, and treatment considerations

**DOI:** 10.1371/journal.pntd.0012136

**Published:** 2024-05-13

**Authors:** Quan Wang, Yanmin Cao, Xinyu Liu, Yaqun Fu, Jiawei Zhang, Yeqing Zhang, Lanyue Zhang, Xiaolin Wei, Li Yang

**Affiliations:** 1 School of Public Health, Peking University, Beijing, China; 2 Brown School, Washington University in St Louis, St Louis, Missouri, United States of America; 3 Jinan Municipal Center for Disease Control and Prevention, Jinan, Shandong Province, China; 4 Centre for Global Health Economics, University College London, London, United Kingdom; 5 Dalla Lana School of Public Health, University of Toronto, Toronto, Ontario, Canada; University of Kelaniya Faculty of Medicine, SRI LANKA

## Abstract

**Background:**

Tuberculosis (TB) and COVID-19 co-infection poses a significant global health challenge with increased fatality rates and adverse outcomes. However, the existing evidence on the epidemiology and treatment of TB-COVID co-infection remains limited.

**Methods:**

This updated systematic review aimed to investigate the prevalence, fatality rates, and treatment outcomes of TB-COVID co-infection. A comprehensive search across six electronic databases spanning November 1, 2019, to January 24, 2023, was conducted. The Joanna Briggs Institute Critical Appraisal Checklist assessed risk of bias of included studies, and meta-analysis estimated co-infection fatality rates and relative risk.

**Results:**

From 5,095 studies screened, 17 were included. TB-COVID co-infection prevalence was reported in 38 countries or regions, spanning both high and low TB prevalence areas. Prevalence estimates were approximately 0.06% in West Cape Province, South Africa, and 0.02% in California, USA. Treatment approaches for TB-COVID co-infection displayed minimal evolution since 2021. Converging findings from diverse studies underscored increased hospitalization risks, extended recovery periods, and accelerated mortality compared to single COVID-19 cases. The pooled fatality rate among co-infected patients was 7.1% (95%CI: 4.0% ~ 10.8%), slightly lower than previous estimates. In-hospital co-infected patients faced a mean fatality rate of 11.4% (95%CI: 5.6% ~ 18.8%). The pooled relative risk of in-hospital fatality was 0.8 (95% CI, 0.18–3.68) for TB-COVID patients versus single COVID patients.

**Conclusion:**

TB-COVID co-infection is increasingly prevalent worldwide, with fatality rates gradually declining but remaining higher than COVID-19 alone. This underscores the urgency of continued research to understand and address the challenges posed by TB-COVID co-infection.

## Author summary

Tuberculosis (TB) and COVID-19, both highly infectious diseases, have posed significant global health challenges, particularly in low/middle-income countries (LMICs) with limited medical resources. Our research highlights that TB-COVID co-infection remains a substantial concern, impacting regions with varying TB burdens. The predominant treatment approach for TB-COVID co-infection has not notably evolved since our earlier study in 2021. It typically involves a combination of the recommended TB regimen and standard COVID-19 treatment. Our analysis consistently shows that individuals with TB-COVID co-infection are at heightened risk of hospitalization, protracted recovery periods, and accelerated mortality compared to those with sole COVID-19 infections. Remarkably, we found limited information on the post-COVID-19 condition of co-infected patients. One study indicated a higher prevalence of anxiety symptoms, highlighting the potential psychological toll of TB-COVID co-infection. Although the fatality rate has gradually decreased, it remains notably higher than that of COVID-19 alone. Our findings underscore the urgent need for global collaboration to address the complex challenges posed by TB-COVID co-infection, particularly in countries with limited medical resources.

## Introduction

The ongoing COVID-19 pandemic has created an unprecedented healthcare crisis, especially in low/middle-income countries (LMICs) where medical resources are severely limited [1,2]. Unfortunately, these countries are also heavily burdened by tuberculosis (TB), with their populations being the main victims of this disease [3]. World Health Organization (WHO) has emphasized that the COVID-19 pandemic has not only disrupted TB services and response but also reversed years of progress made in the fight against tuberculosis [4,5]. Consequently, more people have fallen ill with TB and experienced higher mortality rates, timely diagnosis rates have decreased, and global spending on essential TB services has significantly declined [6].

A systematic review, encompassing evidence from 2019 to mid-2021, revealed a consistent upward trend in the absolute number of co-infected patients. Furthermore, an increasing number of countries reported co-infected patients, including both high-income countries and LMICs [7]. TB, as one of the world’s deadliest infectious diseases, comes second only to COVID-19 in terms of its impact[8]. Some experts believe that TB-COVID co-infection is associated with a poorer prognosis and a higher risk of mortality[9, 10]. It is crucial to note that despite an exhaustive review, we did not encounter a universally accepted definition for TB–COVID co-infection. In this context, our systematic analysis provides a preliminary characterization, defining TB–COVID co-infection as a state arising from both ongoing and past infections involving *M*. *tuberculosis* and SARS-CoV-2. It’s essential to emphasize that while latent TB infection and TB disease (or active TB) present significant clinical distinctions, our usage of ’TB’ in this study encompasses all forms of *M*. *tuberculosis* infection, spanning latent, active, cured, and current states.

While there have been studies that have synthesized evidence on co-infection, they have primarily relied on case reports and case series, providing relatively weak support for epidemiology and treatment [11,12]. Consequently, there remains a dearth of information regarding the treatment and outcomes of TB-COVID co-infection, and a lack of consensus regarding its epidemiological status. This study serves as an update to our previous systematic review, which collected and pooled evidence as of the middle of 2021[7]. In this updated systematic review, we aim to summarize the latest epidemiological data on TB-COVID co-infection, discuss fatality rates, and explore possible clinical outcomes.

## Method

This systematic review follows the PRISMA guidelines (S1 Table) [13]. The study was registered in PROSPERO’s database with the registration number CRD42021253660.

### Search strategy

We conducted a comprehensive search using six electronic databases: MEDLINE, Web of Science, ProQuest, Scopus, Cochrane database, and Embase. To maximize the scope of our search, we also employed the Grey Matters Checklist to identify relevant grey literature [14]. The literature search was conducted until January 24, 2023. Medical Subject Heading (MeSH) terms, title/abstract, topic, or subject words were used in the selected databases. The search formula included the terms "TB" AND "COVID-19". For "TB," key terms such as "tuberculosis," "TB," "tuberculos*," "mycobacterium tuberculosis," and "m.tuberculosis" were used. For "COVID-19," the key terms used were "COVID-19" and "SARS-COV-2".

### Eligibility criteria of included studies

This systematic review included epidemiological and fatality data on TB-COVID co-infection from cohort studies, cross-sectional studies, and experimental research, excluding case reports, series, reviews, editorials, and clinical guidelines. Studies with sample sizes less than 20 were also excluded to reduce potential bias. Two reviewers (QW and XL) independently screened and selected studies using Covidence. Non-English and non-Chinese articles were translated to English using TranslateGo (Hangzhou Qingxun Science and Technology Co., China). Manual reference screening ensured study inclusivity. Conflicts were resolved by a third author (LY), and duplicates were managed across similar studies. We would like to stress that, unlike our previous work in 2021, we did not include case reports or case series in this study. Building on the insights from our earlier research, we found that these study types contributed little to our understanding of the topic, and they did not provide sufficient data for estimating fatality rates, prevalence status, or determining best practices in treatment.

### Data extraction, quality assessment, and analysis

Relevant data, including authors, publication dates, study design, location, sample size, settings, epidemiological and treatment information, and clinical outcomes, were extracted. Prevalence rates of co-infection were prioritized for epidemiological data, along with total and hospitalized fatality rates. The total fatality rate represents the proportion of patients documented as deceased among all TB-COVID co-infected individuals, irrespective of whether they received treatment. On the other hand, the hospitalized fatality rate pertains to the proportion of patients documented as deceased among all TB-COVID co-infected individuals who underwent hospitalization. Treatment details, including drugs and ICU utilization, were also collected. The quality of included studies was evaluated using the Joanna Briggs Institute (JBI) Critical Appraisal Checklist for Study Reporting Prevalence Data [15].

Location data from all studies identified the reporting countries and regions of TB-COVID co-infection cases. Prevalence and fatality rates were chronologically listed for temporal trends analysis.

Random-effects meta-analysis calculated pooled fatality rates and relative risks (RR) of fatality between TB-COVID co-infection and single COVID-19 patients. Forest plots displayed point estimates and 95% confidence intervals (CIs), while *I*^*2*^ assessed heterogeneity. *P* values < 0.05 indicated statistical significance.

Egger’s tests assessed publication bias, and sensitivity analyses assessed robustness by omitting studies one at a time. Subgroup analyses explored LMICs vs. high-income countries and active TB vs. previous TB status. Stata 17 (StataCorp LLC, USA) performed calculations.

## Results

**Fig 1:**
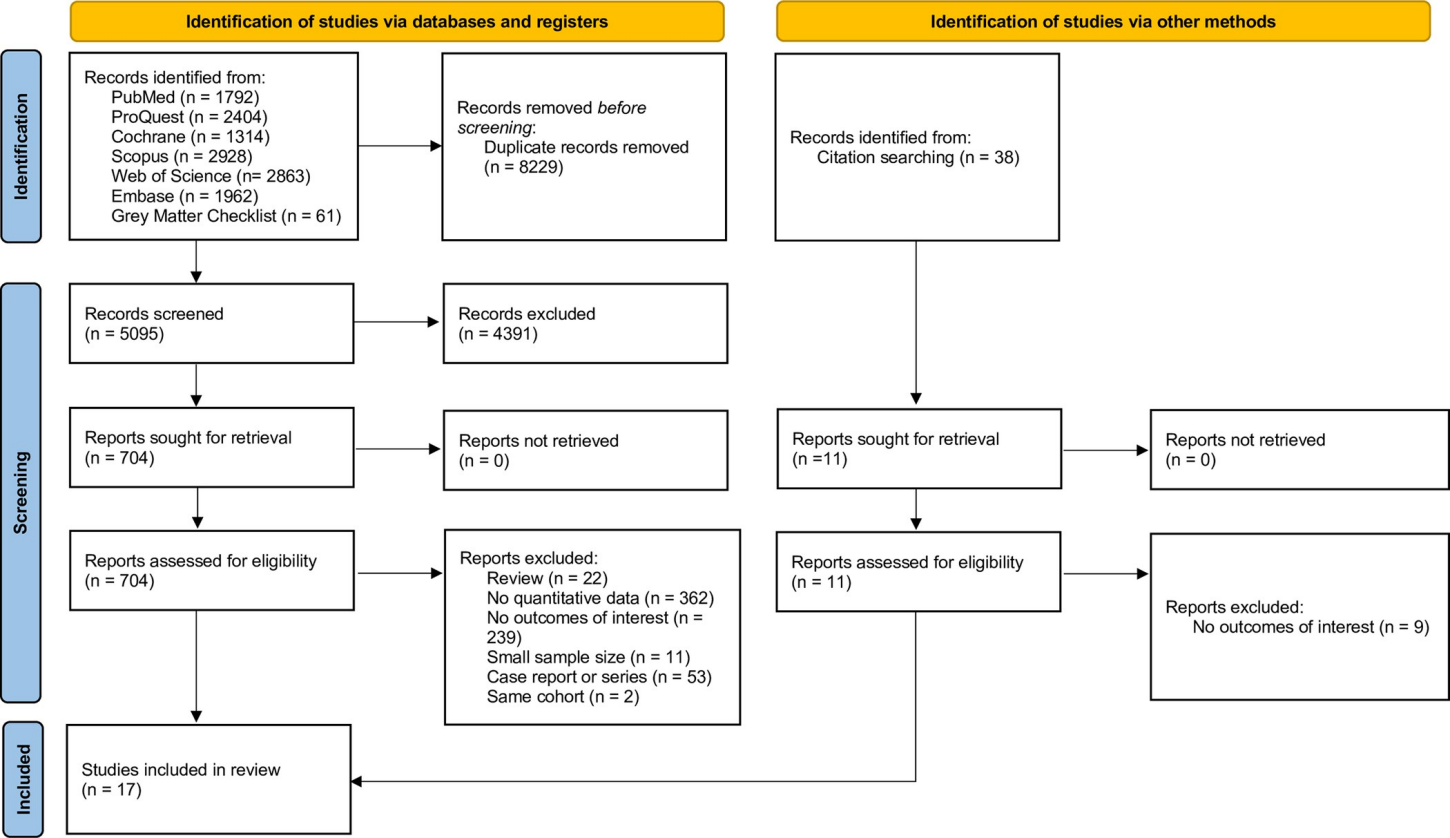
The results of included searches of databases.

A comprehensive search strategy utilizing the building blocks approach was executed to identify pertinent studies. After an extensive search, we retrieved 1,792 records from MEDLINE, 2,863 from Web of Science, 2,404 from ProQuest, 2,928 from Scopus, 1,314 from the Cochrane database, 1,962 from Embase, and 61 from Grey Matters Checklist (refer to S2 Table for details). Upon importing these records into Covidence, 8,229 duplicate records were identified and subsequently removed, resulting in 5,095 records available for title and abstract screening. In this phase, 4,391 records were excluded. The remaining 704 records entered the full-text review process, during which 38 potentially relevant records were identified. Ultimately, 689 out of 704 records and 36 out of 38 records were excluded, and 17 retrospective studies were included for analysis; no experimental studies were identified in the search. The entire process is visually presented in Fig 1.

### Prevalence

As of the search date, our analysis identified TB-COVID co-infection cases reported in 38 countries or regions, including Argentina, Belarus, Belgium, Brazil, Chile, China, France, Republic of Guinea, India, Italy, Mexico, Niger, Pakistan, Panama, Peru, Philippines, Portugal, Romania, Russia, Singapore, Spain, Switzerland, UK, Australia, Canada, Colombia, Greece, Honduras, Lithuania, the Netherlands, Oman, Paraguay, Serbia, Slovakia, South Africa, Turkey, Thailand, and USA. Among the studies included, there was one notable study conducted by the TB/COVID-19 Global Study Group in 2022, which involved TB-COVID patients from 172 centers in 34 countries. The remaining 16 studies reported patients within a single region or country [16].

Regarding the prevalence rate of TB-COVID co-infection, two studies provided information. The first study, conducted by the Western Cape Department of Health in collaboration with the National Institute for Communicable Diseases, analyzed data from the Western Cape Provincial Health Data Centre. They found a prevalence rate of approximately 0.04% among individuals aged 20 years or above in the Western Cape Province until 1 June 2020. After testing criteria changed, the prevalence rate increased to approximately 0.06% until 9 June 2020 [17]. The second study, led by Nabity in 2021, identified 6371 co-infected patients among all California residents between September 3, 2019, and December 31, 2020, resulting in a prevalence rate of approximately 0.02% [18]. The S3 Table provides detailed information on these two studies.

### Treatment

Among the studies included in our analysis, only a limited number of studies provided information on the treatment of TB-COVID co-infection. Upon comparing our findings with our previous study conducted in 2021, we did not identify any new treatments that have emerged. The most commonly utilized treatment approach involved the use of first-line anti-TB treatment (ATT) drugs, including rifampicin, isoniazid, ethambutol, and pyrazinamide, which were administered in the majority of cases. In terms of antiviral drugs, lopinavir, ritonavir, and arbidol were the three most frequently prescribed medications. Notably, the use of hydroxychloroquine (HCQ) has become limited, as it has been demonstrated to have no benefit in the treatment of TB-COVID co-infection [19]. Three studies included in our review highlighted the utilization of Intensive Care Units (ICUs) in the management of TB-COVID co-infection. The reported ICU admission rates varied from 1.3% to 31.8% [20–22]. Additionally, Wang discussed the usage of Paxlovid, an antiviral therapeutic for COVID-19 treatment, and emphasized its contraindication in patients receiving rifampicin, one of the first-line agents for TB treatment, due to drug interactions as Paxlovid is a strong cytochrome P450 3A4 inhibitor. Consequently, Paxlovid was not deemed suitable for treating patients with active TB-COVID co-infection undergoing ATT [21].

Several studies have indicated that TB-COVID co-infected patients face increased risks of hospitalization, longer time-to-recovery in elderly patients, and shorter time-to-death compared to individuals with single COVID-19 infection [21,23–25]. Parolina’s study highlighted various factors associated with an increased risk of developing severe COVID-19 in TB patients, including female gender, smoking, fever, dyspnea, disseminated TB, having three or more co-morbidities, and patient age[26]. Wang emphasized that despite the milder nature of infections with the Omicron variant compared to earlier variants, patients with TB-COVID co-infection do not exhibit the mild disease course observed in the general population [21]. Notably, the majority of patients in Wang’s study, 142 out of 153 co-infected individuals, were classified as nonsevere, with 10 being asymptomatic [21]. This may be attributed to lung parenchyma damage resulting from pulmonary remodeling due to persistent cavitation, fibrosis, or bronchiectasis, which is present in approximately 50% of cured TB patients and may increase susceptibility to COVID-19 and mortality rates [25]. The presence of dual lung damage following both TB and COVID-19 necessitates careful follow-up of patients with post-tuberculosis lung disease who have experienced COVID-19 pneumonia [25]. These findings underscore the complex interactions and challenges associated with TB-COVID co-infection. The coexistence of two lung diseases can lead to heightened severity and poorer outcomes, warranting specialized management approaches and continued monitoring of affected individuals. For more detailed information, please refer to S4 Table.

### Fatality rate

A total of 17studies were included in our analysis, reporting data on the fatality rate of TB-COVID co-infection. The reported fatality rates among the total patient population varied widely, ranging from 0% to 23.6%. Similarly, the in-hospital fatality rates also showed considerable variation, ranging from 0% to 27.3% (Table 1).

**Table 1:** The total/in-hospital TB-COVID patient fatality rate.

First author (year)	Country	Time	Sample size	Active TB (%)	Died	Fatality rate (%)
**Total TB-COVID patients**						
Sy 2020 [24]	Philippines	May 17, 2020 to June 15, 2020	106	100	25	23.6
Davies 2021 [17]	South Africa	Till Mar. 1st 2020	total: 2128	/	113	5.3
Nabity 2021 [18]	U.S.	September 3, 2019, to December 31, 2020	Cohort A: 6280	99	631	10.0
			Cohort B: 91 (less than 120 days TB and COVID-19 diagnoses interval)	95	15	16.5
The GTN 2022 [16]	172 centers from 34 countries	March 2020-June 2021	767	/	85	11.1
	Europe		289	/	41	14.2
	outside Europe		478	/	42	9.2
Otlu 2022 [22]	Turkey	January 1, 2015 to September 31, 2021	71	11	1	1.4
Kayali [27] 2022	Turkey	January 2015 to April 2021	105	97	0	0.0
**Hospitalized TB-COVID patients**						
Gupta 2020 [20]	India	1 February 2020 to 14 June 2020	22	59.1	6	27.2
Sy 2020 [24]	Philippines	May 17, 2020 to June 15, 2020	66	100	18	27.3
Stochino 2020 [28]	Italy	/	20	100	1	5.0
Davies 2021 [17]	South Africa	Till Mar. 1st 2020	469	/	102	21.7
Domingo 2020 [29]	Argentina	March to June, 2020	23	81	2	8.7
Gubkina 2020 [30]	Russia	March to June, 2020	24	100	0	0.0
Hassan 2023 [23]	Pakistan	February 2022—August 2022	218	100	51	23.4
Parolina 2022 [26]	Russian	October 2020 to August 2021	75	100	7	9.3
Sereda 2022 [31]	Belarus	April -October, 2021	47	100	1	2.1
Wang 2022 [21]	Changchun, China	March 2022 to June 2022	153	84	0	0.0
Adzic-Vukicevic 2022 [25]	Serbia	6 March 2020 to 1 April 2022	53	100	1	1.9
Siranart 2023 [32]	Thailand	March 2020 to March 2022	26	/	1	3.8
Malashenkov 2021 [33]	Russia	/	Cohort A: 63	100	13	20.6
			Cohort B: 30	100	3	10.0

/: No information provided

To further explore the impact of active TB and previous TB on fatality rates, we collected and analyzed information specific to these subgroups (Table 2). Among co-infected patients with concurrent TB disease (active TB), the reported fatality rates ranged from 7.6% to 23.6% for the total patient population, and for hospitalized active TB-COVID patients, the fatality rates ranged from 0% to 27.3%. Regarding previous TB-COVID patients, the fatality rates ranged from 4.9% to 14.5% for the total patient population, and for hospitalized patients, the fatality rates ranged from 0% to 24.0%. Please refer to S4 Table and S5 Table, and S6 Table for comprehensive and detailed information about the included studies.

**Table 2:** The active/previous TB-COVID patient fatality rate.

First author (year)	Country	Time	Sample size	Died	Fatality rate (%)
**Active TB-COVID co-infection**					
Davies 2021 [17]	South Africa	Till Mar. 1st 2020	total: 343 in-hospital: 148	total: 26 in-hospital: 25	7.6 (total) 16.9 (in-hospital)
Sy 2020 [24]	Philippines	May 17, 2020 to June 15, 2020	total: 106 in-hospital: 66	total: 25 in-hospital: 18	23.6 (total) 27.3 (in-hospital)
Stochino 2020 [28]	Italy	/	in-hospital: 20	in-hospital: 1	5 (in-hospital)
Parolina 2022 [26]	Russia	October 2020 to August 2021	in-hospital: 75	in-hospital: 7	9.3% (in hospital)
Malashenkov 2021 [33]	Russia	/	in-hospital: 30	in-hospital: 3	10% (in hospital, exclude HIV)
Adzic-Vukicevic 2022 [25]	Serbia	6 March 2020 to 1 April 2022	in-hospital: 53	in-hospital: 1	1.9% (in hospital)
Hassan 2023 [23]	Pakistan	February 2022—August 2022	in-hospital: 218	in-hospital: 51	23.39% (in hospital)
Wang 2022 [21]	China	March 2022 to June 2022	in-hospital: 129	in-hospital: 0	0% (in hospital)
**Previous TB-COVID co-infection**					
Davies 2021 [17]	South Africa	Till Mar. 1st 2020	total: 1785 in-hospital:321	total: 87 in-hospital: 77	4.9 (total) 24.0 (in-hospital)
The GTN 2022 [16]	172 centers from 34 countries	March 2020-June 2021	total: 234	total:34	14.5 (total)
Wang 2022 [21]	China	March 2022 to June 2022	in-hospital: 24	in-hospital: 0	0 (in hospital)

/: No information provided

### Quality assessment of included studies

We employed the JBI Critical Appraisal Checklist for Study Reporting Prevalence Data as a tool to assess the quality of the 17 included studies. The checklist consisted of 9 questions covering various aspects such as sampling method, sample size, study subjects and setting, analysis method, and participant response. Each question was evaluated using one of the four options: yes, no, unclear, or not applicable. In total, 10 studies reached more than 70% of ‘yes’ scores, 6 studies reached from 50% to 69% of ‘yes’ scores, and 1 study was below 50%. Upon further analysis, it was identified that the sample frame, sampling method, and sample size were the areas most frequently identified as having a higher risk of bias within the included studies. Check S7 Table and S1 Fig for assessment result of each study.

### Meta-analysis of fatality rates

**Fig 2:**
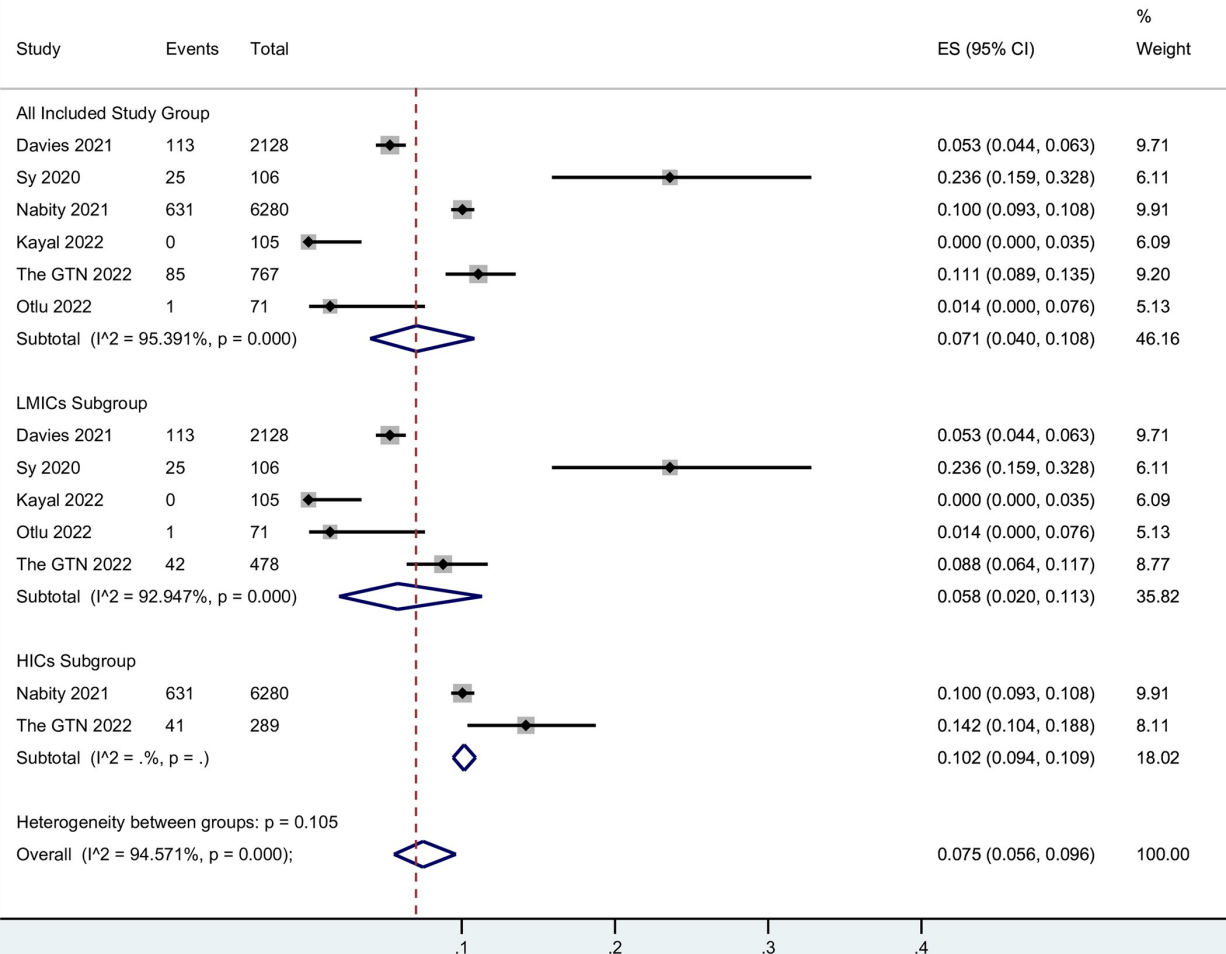
Fatality rate of total TB-COVID co-infection patients.

Among all included studies, the pooled fatality rate of TB-COVID co-infection among total patients was estimated to be 7.1% (95% CI, 4.0%-10.8%). However, when examining the results by country income status, significant variations were observed. In high-income countries (HICs), the pooled fatality rate was higher, with a result of 10.2% (95% CI, 9.4%-10.9%) based on two studies that included a total of 6,569 individuals. On the other hand, in low- and middle-income countries, the pooled fatality rate was lower at 5.8% (95% CI, 2.0%-11.3%), based on five studies involving 2,888 individuals (Fig 2). The GTN’s study provided three cohorts: total co-infected patients, co-infected patients in Europe, and co-infected patients outside of Europe. Considering that most included countries in Europe are HICs and most countries outside of Europe are LMICs, we placed these two cohorts in the HICs and LMICs subgroups, respectively. The results of Egger’s test indicated no evidence of publication bias across all the included study groups, as well as within the low- and middle-income countries subgroup (S8 Table and S2 Fig). To assess the robustness of our pooled results, we performed sensitivity analyses by systematically omitting one study at a time. These analyses consistently demonstrated the stability and reliability of our pooled estimates (S9 Table and S3 Fig).

**Fig 3:**
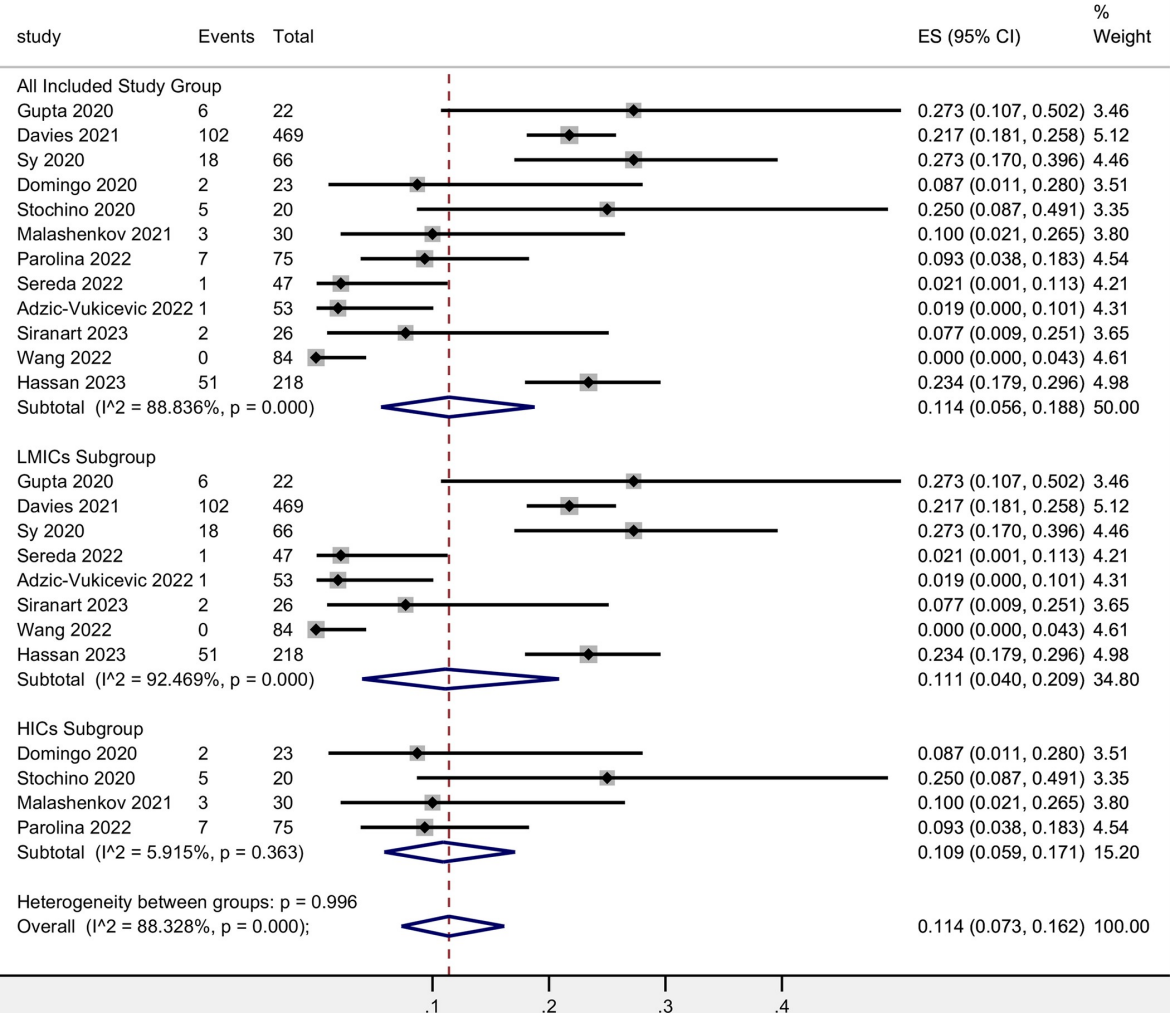
Fatality rate of hospitalized TB-COVID co-infection patients.

The estimated fatality rate among hospitalized patients with TB-COVID co-infection was 11.4% (95% CI, 5.6%-18.8%). It is important to note that significant heterogeneity was detected among the studies and groups analyzed. Unlike the total fatality rate, the results for low- and middle-income countries (LMICs) were similar to those of high-income countries (HICs) in terms of fatality rate among hospitalized patients. The pooled result for LMICs was 11.1% (95% CI, 4.0%-20.9%) based on eight studies involving 985 individuals. In comparison, the pooled result for HICs was 10.9% (95% CI, 5.9%-17.1%) based on four studies involving 148 individuals. These findings suggest a comparable fatality rate among hospitalized TB-COVID co-infection patients in both LMICs and HICs. For detailed results, please refer to Fig 3. Based on the results of Egger’s tests, publication bias was observed in all included study groups. However, no evidence of publication bias was found within the 2 subgroups (S10 Table and S4 Fig). Furthermore, the sensitivity analysis, which involved systematically omitting one study at a time, demonstrated that the exclusion of any particular study did not significantly alter the pooled results. This finding supports the robustness and reliability of our study findings (S11 Table and S5 Fig).

**Fig 4:**
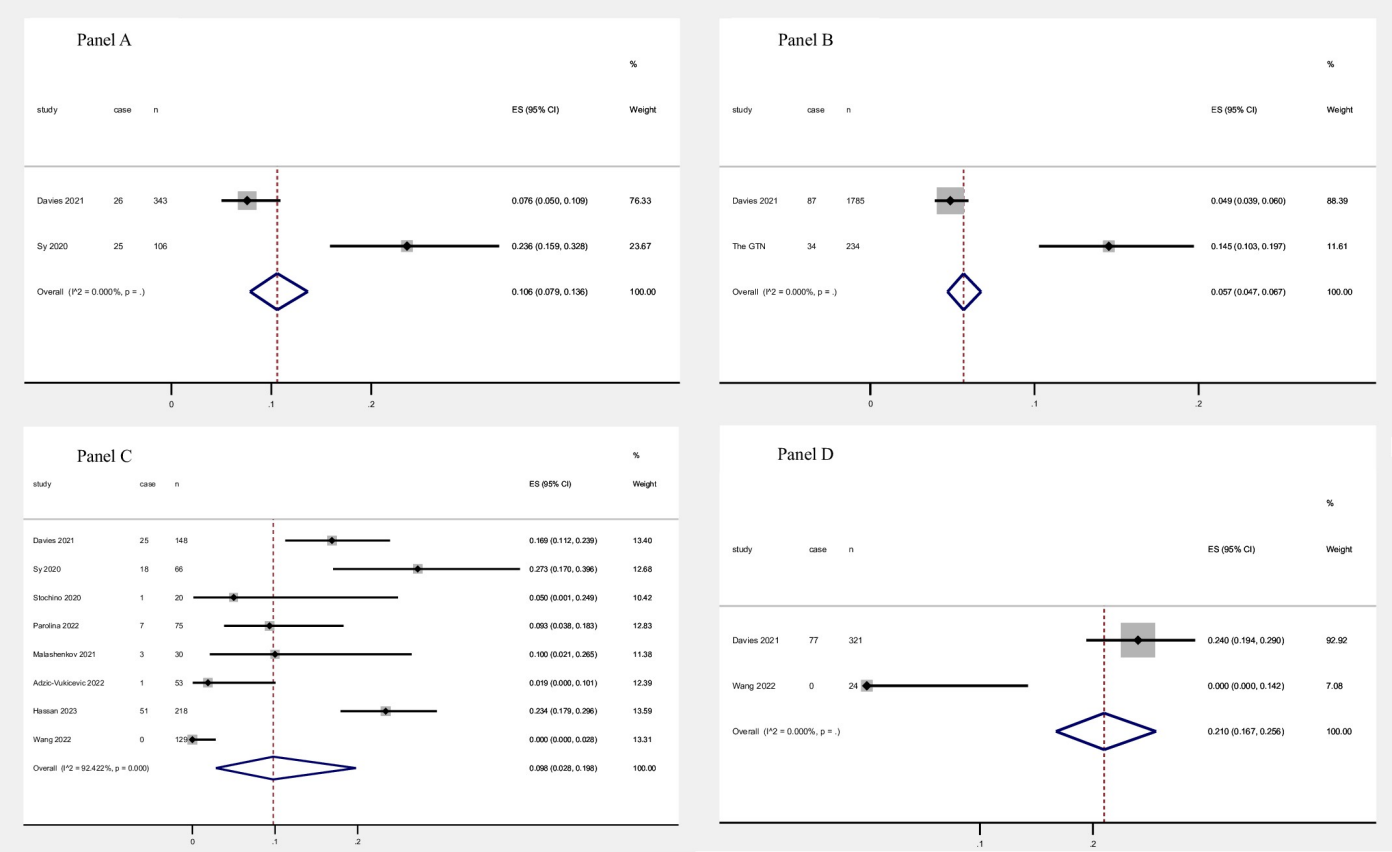
Fatality rate of active/previous TB-COVID co-infection patients. A: Total active TB-COVID co-infection patients; B: Total previous TB-COVID co-infection patients; C: Hospitalized active TB-COVID co-infection patients; D: Hospitalized previous TB-COVID co-infection patients.

In our subgroup analysis based on TB status (active/previous), the pooled results revealed significant differences in the fatality rates between active TB-COVID infection and previous TB- COVID infection (Fig 4). For total fatality rate, the pooled estimate for active TB-COVID infection was 10.6% (95% CI, 7.9%-13.6%), which was higher compared to previous TB-COVID infection with a pooled estimate of 5.7% (95% CI, 4.7%-6.7%). Regarding in-hospital fatality rate, the estimated pooled result for active TB-COVID infection was 9.8% (95% CI, 2.8%-19.8%) based on eight studies involving 739 individuals. In contrast, the in- hospital fatality rate for previous TB-COVID infection was higher, with a pooled estimate of 21.0% (95% CI, 16.7%-25.6%). Furthermore, Egger’s tests were conducted to assess publication bias, and the results can be found in S12 Table and S6 Fig. Additionally, sensitivity analyses were performed, and the results demonstrated the stability and robustness of the study findings (refer to S13 Table and S7 Fig).

### Meta-analysis of relative risk

Three studies included in our analysis provided results on the relative risk (RR) of in-hospital fatality between TB-COVID patients and single COVID patients. The pooled analysis, which involved a total of 1285 patients, suggested that TB infection might potentially reduce the fatality risk with a relative risk estimate of 0.8 (95% CI, 0.18–3.68) (S8 Fig). Regarding publication bias, the results of Egger’s test indicated no evidence of publication bias in the included studies (S9 Fig). However, the sensitivity analysis revealed some instability in the pooled results, suggesting the need for caution in interpreting these findings (S14 Table and S10 Fig).

## Discussion

This updated systematic review collected relevant studies up until January 24, 2023, and included a total of 17 studies. In comparison to a previous study that identified co-infection cases in 12 countries or regions based on population studies, our review expanded the scope and identified an additional 18 countries or regions reporting TB-COVID co-infection. This finding suggests that despite COVID-19 no longer being classified as a Public Health Emergency of International Concern by the WHO, the prevalence of TB-COVID co-infection remains significant in both high and low TB-burden countries or regions.

A notable contribution to the field is a large-scale study led by Nabity in 2021, which provided an updated prevalence rate estimate of approximately 0.02% in California, USA [18]. In contrast, an earlier study conducted in West Cape Province, South Africa in early 2020 reported a prevalence rate of 0.06%. The discrepancy in prevalence rates between these two regions suggests that the burden of TB-COVID co-infection may vary across different geographic locations. Factors such as differences in TB prevalence, COVID-19 incidence, and the effectiveness of TB and COVID-19 control measures implemented in each region may contribute to these variations.

In terms of treatment, our analysis revealed that the treatment approach for TB-COVID co-infection has not undergone significant changes since our previous study in 2021. The predominant strategy employed in the included studies involved the administration of first-line anti-TB drugs, which is in accordance with the established standard treatment protocol for TB. Despite our comprehensive review of the available literature, we did not identify any experimental studies that could provide specific guidance on the best practices for managing TB-COVID co-infection. Only a limited number of studies made any mention of adjustments to treatment regimens based on the unique characteristics of co-infected patients. As a result, the current approach to treatment for TB-COVID co-infection appears to be a combination of the recommended TB regimen and the standard treatment for COVID-19.

The studies that reported ICU utilization in the context of TB-COVID co-infection provided insights into the severity of the disease and the clinical management required. The wide range of reported ICU admission rates, ranging from 1.3% to 31.8%, highlights the heterogeneity in disease presentation and underscores the need for specialized care for individuals with severe forms of co-infection. These findings emphasize the importance of tailored management approaches that address the complex interactions between TB and COVID-19. Consistent findings across multiple studies indicate that individuals with TB-COVID co-infection face a higher risk of hospital admission, longer time-to-recovery, and shorter time-to-death compared to individuals with single COVID-19 infection [21, 23–25]. These observations underscore the unique challenges posed by the coexistence of TB and COVID-19 and emphasize the necessity for tailored management strategies that effectively address both diseases.

Another important aspect to consider in the context of TB-COVID co-infection is the potential development of Post-COVID-19 condition (PCC), commonly known as long COVID [34]. PCC refers to a range of persistent symptoms and health issues that can affect individuals even after recovering from acute COVID-19 infection [35]. It has been observed that PCC can significantly impact a person’s daily functioning, employability, and overall well-being. Moreover, it has been associated with an increased risk of developing new health conditions and the utilization of healthcare services, which can further strain the individual’s financial stability [34]. However, it is worth noting that the current evidence regarding PCC specifically in the context of TB-COVID co-infection is scarce. We only identified one study that mentioned the proportion of individuals experiencing long-lasting symptoms after COVID-19 infection in conjunction with previous tuberculosis (PTB) treatment [36]. This study reported that over time, the proportion of individuals with persistent symptoms decreased, although a significant proportion, approximately one in six, still experienced ongoing symptoms. Furthermore, this group exhibited a higher prevalence of anxiety symptoms, underscoring the potential psychological impact of TB-COVID co-infection. The recurrence of pulmonary tuberculosis and the need for psychological support for individuals with a history of both COVID-19 and pulmonary TB after discharge warrant additional attention and investigation [36].

The meta-analyses conducted on the overall fatality rate of TB-COVID co-infection revealed an estimated rate of 7.1%, which is lower than our previous study’s estimate of 13.9%. This difference could potentially be attributed to the emergence of new SARS-CoV-2 variants that may exhibit milder clinical manifestations. However, it is important to note that the fatality rate of TB-COVID co-infection remains higher than that of COVID-19 alone, which was estimated at 0.68% by mid of 2020[37]. Subgroup analyses based on high-income countries and low- and middle-income countries showed a higher fatality rate in high-income countries (10.2%) compared to LMICs (5.8%). It is crucial to recognize that multiple confounding factors may contribute to this observed discrepancy. For instance, lower vigilance and delayed time-to-diagnosis in outpatient clinics, particularly in higher-income countries with traditionally lower TB burdens, could play a role. Another potential factor is the higher frequency of COVID-19 testing in high-income countries, which might dilute the numbers of identified active TB-COVID infection. Additionally, the average age of co-infected patients tends to be higher in HICs, and given that age is a proven risk factor for COVID-19 mortality, this demographic difference could contribute to the observed higher fatality rate. These findings underline the importance of considering various contextual factors when interpreting fatality rates and emphasize the need for further research to elucidate the complex dynamics at play. In terms of in-hospital fatality rates, the results were similar between high-income countries (11.1%) and LMICs (10.9%), further supporting the assumption mentioned above.

Our subgroup analysis based on TB status (active/previous) revealed significant differences in the fatality rates between active TB-COVID infection and previous TB-COVID infection. These findings highlight the differential risks and outcomes associated with active and previous TB in the context of COVID-19 co-infection. The reasons for these differences may be multifactorial. Active TB-COVID infection may impose a greater burden on the immune system and respiratory function, leading to increased susceptibility to severe COVID-19 illness and poorer outcomes. In contrast, individuals with previous TB may have partially developed immunity or residual lung damage, which could potentially confer some level of protection or adaptation against severe COVID-19. We acknowledge the variability in the status of TB infection extracted from the included origin studies, as there was no uniform standard criterion across different studies. Active TB is a complex disease with a lengthy treatment regimen, which is commonly defined as disease that occurs in someone infected with *Mycobacterium tuberculosis*. It is characterized by signs or symptoms of active disease, or both, and is distinct from latent tuberculosis infection, which occurs without signs or symptoms of active disease [38]. The absence of consistent definitions or criteria may have contributed to the heterogeneity observed in the meta-analysis.

An intriguing trend in current TB-COVID research centers around a significant focus on the pandemic’s impact on TB care services. Global studies have demonstrated a substantial adverse effect on the delivery, accessibility, and utilization of TB care services [39]. Comparing 2020 to 2019, there was an 18% reduction in global tuberculosis case detection, dropping from 7.1 million to 5.8 million cases, with up to a 24% decrease in the ten worst-affected countries with a high tuberculosis burden [5]. This service disruption in TB care has led to a consequential increase in additional tuberculosis-related deaths. From a critical thinking perspective, we posit that this impact might contribute to an augmentation in our estimated TB-COVID fatality rate in two crucial ways. Firstly, the reduction in tuberculosis case detection may result in fewer identified TB-COVID co-infected patients. This is particularly significant as COVID-related deaths are usually more rigorously recorded in many countries, and during this process, the TB infection can also be documented. Secondly, the disruption in TB care services might result in insufficient treatment for numerous co-infected individuals, potentially contributing to preventable deaths. This concern is particularly pronounced in LMICs, where healthcare services are often limited and of lower quality [40, 41]. Additionally, the decrease in discovered cases of TB could contribute to a lower total number of identified co-infected patients.

In our analysis, we observed a relative risk (RR) value suggesting that TB-COVID co-infection might reduce the fatality risk compared to single COVID-19 infection. This finding may initially seem counterintuitive given that TB is a known risk factor for severe respiratory illness and mortality. It’s essential to emphasize that the groups with TB-COVID co-infection and those with single COVID-19 infection did not exhibit precisely homogeneous patient characteristics, including differences in age, gender, comorbidities, and treatment modalities. For instance, studies by Parolina and Sereda reported a higher proportion of male patients in the TB-COVID co-infection group compared to the single COVID-19 infection group [26, 31]. Also of note is that Sy’s 2020 study, employing propensity score matched sampling, suggested that co-infected patients experienced higher fatality rates [24]. However, due to the limited information available regarding the specific details of the included patient groups, we cannot deduce the underlying reasons for this counterintuitive RR. Therefore, readers are advised to approach this finding with caution and interpret it within the acknowledged limitations we have outlined.

As a systematic review focused on TB-COVID co-infection, understanding how TB impacts COVID-19 is as crucial as comprehending how COVID-19 impacts TB. However, given the prominence of COVID-19 as a research topic, many studies at the individual level tend to emphasize the perspective of COVID-19 infection. While we did encounter studies exploring how COVID-19 impacts TB, these primarily delved into microbiological mechanisms or the pandemic’s disruption of TB service delivery. Immunologically, a shared dysregulation of immune responses in COVID-19 and TB has been identified, indicating a dual risk posed by co-infection in worsening COVID-19 severity and favoring TB disease progression [42, 43]. Notably, for some severe COVID-19 patients, corticosteroid use can induce immunosuppression [44], significantly increasing the risk of new secondary infections and/or reactivation of existing quiescent TB infections [45, 46]. From the TB service perspective, the COVID-19 pandemic has substantially impacted the normal delivery of TB services, exerting a negative influence on TB patients [39]. However, some studies suggest a potential reduction in *Mycobacterium tuberculosis* transmission during the pandemic, potentially lowering TB fatality rates [47, 48]. Unfortunately, the current evidence is limited, and the impact of the pandemic on TB remains conflicting and inconclusive. We cautiously posit that COVID-19 exerts a negative influence on individuals already carrying *Mycobacterium tuberculosis*.

In our assessment of study quality, two critical bias factors emerged: insufficient sample size and unappreciated sample frame. Insufficient sample size refers to studies with limited participants, hampering findings’ generalizability. With relatively lower prevalence for TB-COVID co-infection compared to individual TB or COVID-19, obtaining a sizeable co-infected cohort, especially where TB and COVID-19 are rarer, becomes challenging. Limited sample size may curtail statistical power and precision, potentially biasing prevalence estimates. Unappreciated sample frame denotes studies unintentionally selecting populations misrepresenting the target group. Poorly described sampling or inclusion criteria misaligned with intended population characteristics can lead to biases. In TB-COVID co-infection, ensuring representation of individuals with both conditions, not biased subgroups, is vital. Incorrect sample framing may introduce biases and limit findings’ applicability.

While we recognize that a randomized controlled trial (RCT) stands as the gold standard for investigating treatments or risk factors, we contend that diverse study designs can offer valuable contributions to this field. In light of our current findings, we advocate for the consideration of a comparable sampling frame, such as the utilization of propensity score matched sampling in future studies. This approach allows for the creation of balanced groups, resembling the random assignment achieved in an RCT, thus minimizing selection bias and improving the internal validity of observational studies. Furthermore, we propose a more comprehensive description of patients’ baseline conditions and treatment regimens in subsequent research endeavors. This detailed information holds the potential to mitigate bias significantly. A thorough account of patients’ characteristics and treatment variables enhances the ability to control for confounding factors, providing a clearer understanding of the associations under investigation. Employing such strategies not only bolsters the robustness of observational studies but also facilitates the comparability of findings across different research designs.

Several limitations should be acknowledged in the interpretation of our findings. First, we did not include “comorbidity” as a keyword and MeSH term in the searching process, which might have resulted in the omission of relevant studies taking TB as a kind of comorbidity of COVID-19 patients. Second, the observational design precludes establishing causation, and although we employed rigorous statistical methods to control for confounding factors, residual confounders may persist. Third, the generalizability of our results may be influenced by the predominantly retrospective and multicentric nature of the included studies. Variability in healthcare settings, patient populations, diagnostic criteria, and treatment approaches across different regions and countries could impact the external validity of our findings. Additionally, the lack of uniformity in reporting across studies may have introduced inconsistencies in our data synthesis. Furthermore, the limited availability of detailed information on certain variables, such as socioeconomic status, comorbidities, and *M*. *tuberculosis* infection status, restricted our ability to conduct more granular subgroup analyses. As mentioned earlier, distinctions exist among latent, active, cured, and current *M*. *tuberculosis* infections. However, due to insufficient details, we faced considerable challenges in differentiating between these states. Finally, the evolving landscape of the COVID-19 pandemic and variations in healthcare infrastructure over time may have influenced treatment strategies and outcomes. Despite these limitations, our study provides valuable insights into the landscape of TB-COVID co-infection, emphasizing the need for further research to address these complexities comprehensively.

## Conclusion

In conclusion, the fatality rate of co-infection declined gradually and still stayed higher than COVID-19 alone, underscoring the heightened vulnerability in co-infected individuals. Addressing this challenge requires targeted measures such as heightened awareness campaigns, improved screening strategies for TB infection, and the provision of comprehensive long COVID care for co-infected patients. Collaboration on a global scale may be beneficial in addressing the challenges posed by TB-COVID co-infection, particularly in regions with limited medical resources.

## Supporting information

S1 Table The Preferred Reporting Items for Systematic Reviews and Meta-Analyses (PRISMA) 2020 checklist. (PDF)

S2 Table Search strategies to identify studies reporting the prevalence status, treatment and outcomes of tuberculosis and COVID-19. (PDF)

S3 Table Studies reported prevalence rate (n = 2). (PDF)

S4 Table Detailed basic information of included studies (n = 17). Detailed basic information of included case reports (n = 17).
(PDF)

S5 Table The fatality rates of active and previous TB-COVID co-infection (n = 11). The fatality rates of active TB-COVID co-infection (n = 11).
(PDF)

S6 Table The fatality rates of previous TB-COVID co-infection (n = 3). (PDF)

S7 Table Quality assessment of each included study. (PDF)

S8 Table Egger’s test on total fatality rate. (PDF)

S9 Table Sensitives analysis on MA of total fatality rate. (PDF)

S10 Table Egger’s test on MA of In-hospital fatality rate. (PDF)

S11 Table Sensitives analysis on MA of In-hospital fatality rate. (PDF)

S12 Table Egger’s test on MA of active/previous TB-COVID co-infection fatality rate. (PDF)

S13 Table Sensitives analysis on MA of In-hospital fatality rate. (PDF)

S14 Table Sensitives analysis on RR of in-hospital fatality between TB-COVID patients and single COVID patients. (PDF)

S1 Fig Quality assessment of included studies (N = 17). (PDF)

S2 Fig Egger’s test on MA of total fatality rate. (PDF)

S3 Fig Sensitives analysis on MA of total fatality rate. (PDF)

S4 Fig Egger’s test on MA of In-hospital fatality rate. (PDF)

S5 Fig Sensitives analysis on MA of In-hospital fatality rate. (PDF)

S6 Fig Egger’s test on MA of hospitalized Active TB-COVID co-infection patients fatality rate. (PDF)

S7 Fig Sensitives analysis on MA of hospitalized Active TB-COVID co-infection patients fatality rate. (PDF)

S8 Fig Relative risk of in-hospital Fatality between TB-COVID co-infection and Single COVID-19 co-infection. (PDF)

S9 Fig Egger’s test on RR of in-hospital fatality between TB-COVID patients and single COVID patients. (PDF)

S10 Fig Sensitives analysis on RR of in-hospital fatality between TB-COVID patients and single COVID patients. (PDF)
