# Supplementary material for: Systematic review and meta-analysis of Tuberculosis and COVID-19 Co-infection: Prevalence, fatality, and treatment considerations
Source: PLoS Negl Trop Dis. 2024 May 13;18(5):e0012136. doi: 10.1371/journal.pntd.0012136 (PMC11090343; doi:10.1371/journal.pntd.0012136)
Supplement: S2 Table — (PDF) [file pntd.0012136.s002.pdf]

S2 Table Search strategies to identify studies reporting the prevalence status, treatment and outcomes of tuberculosis and COVID-19

Database searched: PubMed

Date: January 24, 2023

Limits: Nov. 1st, 2019 to January 24, 2023

#	Searches	Results
1	("covid-19"[MeSH Terms] OR "sars-cov-2"[MeSH Terms]) AND "tuberculosis"[MeSH Terms]	662
2	((covid-19[Title/Abstract]) OR (Sars-cov-2[Title/Abstract])) AND((TB[Title/Abstract]) OR (tuberculos*[Title/Abstract]))	1701
3	#1 OR #2	1792

Database searched: Web of Science

Date: January 24, 2023

Limits: Nov. 1st, 2019 to January 24, 2023

#	Searches	Results
1	((TS=(covid-19)) OR (TS=(sars-cov-2)) OR (AB=(covid-19)) OR (AB=(sars-cov-2)) OR (TS=(covid-19)) OR (TS=(sars-cov-2)) OR (AB=(covid-19))) AND ((TS=(tuberculosis)) OR (TS=(Mycobacterium tuberculosis)) OR (TS=(Mycobacterium tuberculosis)) OR (AB=(tuberculo*)) OR (AB=(TB)) OR (AB=(Mycobacterium tuberculosis)) OR (AB=(M.tuberculosis)) OR (AB=(tubercle bacillus)))	2863

Database searched: ProQuest

Date: January 24, 2023

Limits: Nov. 1st, 2019 to January 24, 2023

#	Searches	Results
1	su(COVID-19 OR SARS-COV-2) AND su(TB OR tuberculo* OR (Mycobacterium tuberculosis) OR (M.tuberculosis))	2404

Database searched: Scopus

Date: January 24, 2023

Limits: Nov. 1st, 2019 to January 24, 2023

#	Searches	Results
1	(TITLE-ABS-KEY (covid-19 OR sars-cov-2) AND TITLE-ABS-KEY (tb OR tuberculos* OR (mycobacterium AND tuberculosis) OR (m.tuberculosis)))	2928

Database searched: Cochrane database

Date: January 24, 2023

Limits: Nov. 1st, 2019 to January 24, 2023

#	Searches	Results
1	MeSH descriptor: [COVID-19] explode all trees	2590
2	MeSH descriptor: [SARS-COV-2] explode all trees	1190
3	MeSH descriptor: [tuberculosis] explode all trees	2663
4	(#1 OR #2) AND 3	1314

Database searched: Embase

Date: January 24, 2023

Limits: Nov. 1st, 2019 to January 24, 2023

#	Searches	Results
1	('covid 19':ab,ti OR 'sars cov 2':ab,ti)	333,023
2	(tuberculosis:ab,ti OR 'mycobacterium tuberculosis':ab,ti OR tuberculo*:ab,ti OR 'tb':ab,ti OR 'm.tuberculosis':ab,ti OR 'tubercle bacillus':ab,ti)	308,188
3	#1 AND #2	1962

Database searched: Grey Matters Checklist

Date: January 24, 2023

Limits: Nov. 1st, 2019 to January 24, 2023

#	Databases	Searches	Results
1	Bandolier Knowledge	Tuberculosis COVID (Match All Words)	0
2	Bandolier Knowledge	TB COVID (Match All Words)	0
2	McMaster University, McMaster Health Forum	Tuberculosis COVID	2
3	Latin-American and Caribbean Center on Health Sciences Information<br>(LILACS)	((Tuberculosis) OR (COVID)) AND (tax)<br>Tittle, abstract, subject	59
