# Supplementary material for: Systematic review and meta-analysis of Tuberculosis and COVID-19 Co-infection: Prevalence, fatality, and treatment considerations
Source: PLoS Negl Trop Dis. 2024 May 13;18(5):e0012136. doi: 10.1371/journal.pntd.0012136 (PMC11090343; doi:10.1371/journal.pntd.0012136)
Supplement: S3 Table — (PDF) [file pntd.0012136.s003.pdf]

S3 Table Studies Reported Prevalence Rate (n=2)

First author (year)	Country	Study design	Time	Sample size	Gender	Coinfected patients' size	% Active TB
Davies 2021	Western Cape, South Africa	Cross-sectional	Before 1, March	3460932	male 42%	2128	343(16.1%)
Nabity 2021	California, U.S.	Cross-sectional	September 3, 2019, to December 31, 2020	3409084	/	6280	5953 (94.8%)

Please note that the information provided is based on the reported findings by authors and included active/previous TB-COVID infection patients.
