# Supplementary material for: Systematic review and meta-analysis of Tuberculosis and COVID-19 Co-infection: Prevalence, fatality, and treatment considerations
Source: PLoS Negl Trop Dis. 2024 May 13;18(5):e0012136. doi: 10.1371/journal.pntd.0012136 (PMC11090343; doi:10.1371/journal.pntd.0012136)
Supplement: S4 Table — Detailed basic information of included case reports (n = 17). (PDF) [file pntd.0012136.s004.pdf]

S4 Table Detailed basic information of included studies (n=17)

First author (year)	Country	Study design	Single center	Time	Sample size	Age	Gender	Comorbidity	% BCG vaccination
Gupta 2020	India	retrospective observational study	yes	1 February 2020 to 14 June 2020	22	36 (27–59.5)	20 male	/	/
Motta 2020	Belgium, Brazil, France, Italy, Russia, Singapore, Spain, and Switzerland	retrospective observational study	no	/	cohort A: 49<br>cohort B: 20	cohort A: 40(27-49)<br>cohort B: 66(46–70)	/	/	/
Sy 2020	Philippines	retrospective observational study	no	May 17, 2020 to June 15, 2020	total: 106<br>in-hospital :66	total: 50.33(21.68)	48(72.7)	Hypertension 16(24.2)<br>Diabetes 10(15.2)<br>Cancer 1(1.5)<br>Renal cancer 4(6.1)<br>Cardiac disease	/

								7(10.6)<br>Asthma 1(1.5)<br>COPD 1(1.5)	
Stochino 2020	Italy	retrospective observational study	yes	/	20	39(27-47)	12 (60%)	/	3(15%)
Davies 2021	South Africa	retrospective observational study	no	Till Mar. 1st 2020	total: 2128<br>in-hospital :469	/	/	/	/
Domingo 2020	Argentina	retrospective observational study	no	March to June, 2020	in-hospital :23 (4 children)	36.9 (children not included)	18	Smoking and other addictions 16(69.6)<br>HIV 4(17.4)<br>Psychosis 1(4.3)<br>Pulmonary thromboembolism	

								1(4.3) Arterial hypertension 1(4.3)	
Gubkina 2020	Russia	retrospective observational study	yes	March to June, 2020	24 (children)	/	/	/	/
Hassan 2023	Pakistan	retrospective observational study	yes	February 2022 - August 2022	in-hospital :218	/	male: 170 (77.98%)	without-COPD	/
Parolina 2022	Russian	retrospective observational study	2	October 2020 to August 2021	in-hospital :75	45-52	male: 49 (65.3)	Cardiovascular disease 25 (33.3) Chronic respiratory disease 12 (16.0) Chronic liver disease 17 (22.7)	/

								Diabetes mellitus 8 (10.7)<br>Chronic renal disease 5 (6.7)<br>Chronic gastrointestinal tract disease 5 (6.7)<br>Nervous system diseases 13 (17.3)	
Sereda 2022	Belarus	retrospective observational study	7	April - October, 2021	in-hospital :47	/	male 36(77%)	/	/
Nabity 2021	California, U.S.	retrospective observational study	/	September 3, 2019, to December 31, 2020	Cohort A: total: 6280	58 (41.0-73.0)	male 52 (57%)	Diabetes 42 (46.2)<br>HIV 4 (4.9)<br>End-stage kidney disease 5 (5.5)	/

					Cohort B: total: 91 (less than 120 days TB and COVID-19 diagnoses interval)	56 (35.0-70.0)	male 3839 (61%)	Diabetes 1734 (27.6) HIV 197 (3.5) End-stage kidney disease 250 (4.0)	/
The GTN 2022	172 centres from 34 countries (Argentina, Belarus, Belgium, Brazil, Chile, China, France, Republic of Guinea, India, Italy, Mexico, Niger, Panama, Peru, Portugal, Romania, Russia, Singapore, Spain, Switzerland , UK， Australia, Canada, Colombia, Greece,	retrospective observational study	172	March 2020-June 2021	767	44 (31–58)	male 540 (70.4)	COPD 59/751 (7.8) HIV 83/724 (11.5) End-stage kidney disease 250 (4.0) Renal failure 53/713 (7.4) Dialysis 17/43 (39.5) Liver disease 60/700 (8.6)	349/385 (90.7%)

	Honduras, Lithuania, the Netherlands, Oman, Paraguay, Serbia, Slovakia, South Africa and USA.)								
	Europe		/		289	/	/	/	/
	outside Europe		/		478	/	/	/	/
Wang 2022 (Omicron variant)	Changchun, China	retrospective observational study	yes	March 2022 to June 2022	153	53 (15-89)	male 104 (68)	Diabetes 24 (15.7) coronary disease 16 (10.5) hypertension 15 (10.5)	/

Adzic-Vukicevic 2022	Serbia	retrospective observational study	yes	6 March 2020 to 1 April 2022	53	/	male 35 (66)	Cardiac disease 12 (22.6) Respiratory disease 19 (35.8) Diabetes 9 (17.0)	/
Otlu 2022	Turkey	retrospective observational study	/	January 1, 2015 to September 31, 2021	71	11-84	male 31 (43.7)	/	/
Siranart 2023	Thailand	retrospective observational study	yes	March 2020 to March 2022	26	48 (17-85)	male 17 (65.4)	/	/
Malashenkov 2021	St. Petersburg, Russia	retrospective observational study	yes	/	63	44.8 (24-86)	male 47 (74.6)	HIV 30 (53.5)	/

Kayal 2022	Turkey	retrospective observational study	/	January 2015 to April 2021	105	29-67	male 41 (39.05)	Chronic pulmonary diseases 15 (14.29) Chronic renal disease 6 (5.71) Cerebrovascular diseases 3 (2.86) Diabetes mellitus 12 (11.43) Chronic cardiac diseases 29 (27.62) Cancer 7 (6.67) Neurological diseases 3 (2.86) Gastrointestinal system diseases 1 (0.95)	/

Rheumatological diseases 2 (1.90)

S4 Table Detailed basic information of included case reports (n=17)

First author (year)	% pulmonary TB	% extrapulmonary TB	% drug resistance	% active TB	% has TB before COVID-19	% Symptomatic (yes or no)	% ICU	Treatment	Died
Gupta 2020	17(77.3%)	5(22.7)	/	13(59.1%)	/	19(95%)	7(31.8%)	/	6
Motta 2020	/	/	/	/	/	/	/	/	cohort A: 7 cohort B: 1

Sy 2020	/	/	/	100%	/	/	/	/	total: 25 in-hospital: 18
Stochino 2020	19(95%)	3(15%)	5(40%)	100%	/	100%	/	ATT, HQC	1
Davies 2021	/	/	/		total: 343 (16.1%) in-hospital :148(31.6)	/	/	/	total: 113 in-hospital: 102
Domingo 2020	18(78%)	3(13%)	2(9%)	21(81%)	/	/		ATT (standard regimen)19 second-line ATT for patients with MDR or severeserious adverse reaction4	2

Gubkina 2020	/	/	/	100%	100%	14(58.3%)	/	/	0
Hassan 2023	/	/	/	100%	/	100%	/	/	51
Parolina 2022	100%	/	42(56%)	100%	100%	100%	/	/	7
Sereda 2022	/	/	28%(13/47)	100%	/	100%	/	/	1
Nabity 2021	75 (82.4)	28.60%	6.70%	99%	100.00%	100%	/	/	15 died
	5149 (82.0)	30.60%	10.60%	95%	/	100%	/	/	631 died

The GTN 2022	648/755 (85.8)	189/738 (25.6)	90/607 (14.8)	/	553/747 (74.0%)	100%	/	Ventilation and oxygen therapy; Antivirals, Lopinavir/ritonavir,Darunavir/cobicistat or darunavir/ritonavir, Favipiravir, Remdesivir,Other antivirals , Immunomodulators ,Glucocorticoids (methylprednisolone, betamethasone, ciclesonide, other glucocorticoids) ,Intravenous immunoglobulin, IL-6 inhibitors, Bevacizumab (antibody against VEGF-A), Anticoagulants, Enoxaparin, Other therapeutic anticoagulants, Miscellaneous , Azithromycin , Hydroxychloroquine , N-acetyl-cysteine, Plasma from recovered	85

								patients , Interferon ,Other nonsteroidal anti-inflammatory drugs	
	/	/	/	/	/	100%	/	/	41
	/	/	/	/	/	100%	/	/	42
Wang 2022 (Omicron variant)	129 (84.3)	25 (16.3)	12 (7.8)	84%	/	100%	2/153(1.3%)	rifampin, oxygen therapy, Chinese traditional medicine	0
Adzic-Vukicevic 2022	52 (98.0)	1 (2.0)	2 (3.8)	100%	/	100%	/	Oxygen support via high-flow nasal cannula and later noninvasive ventilation; Antiviral	1

								drugs(favipiravir, molnupiravir, and remdesivir); Immunomodulatory treatment; anticoagulation therapy ;azithromycin	
Otlu 2022	/	/	/	11%	8 (11.2%)	/	5(7%)	/	1
Siranart 2023	/	/	/	/	/	100%	/	standard symptomatic care, favipiravir, remdesivir, corticosteroid	1
Malashenkov 2021	51%	23 (36.5)	/	100%	/	100%	/	ATT	13 (10 HIV, 3 no HIV)

Kayal 2022	47 (44.7%)	64 (61.0%)	/	97%	100%	100%	/	First-line drugs: isoniazid, rifampicin, ethambutol, and pyrazinamide. Sensitive ones were used in the treatment.Second-line drugs: ethionamide, prothionamide, cycloserine, and terizidone (since they were similar drugs, one of ethionamide and prothionamide, one of cycloserine and terizidone were used).<br>First-line drugs 97 (97.00)<br>First-line drugs and second-line drugs 3 (3.00)<br>Second-line drugs 0	0
