# Supplementary material for: Systematic review and meta-analysis of Tuberculosis and COVID-19 Co-infection: Prevalence, fatality, and treatment considerations
Source: PLoS Negl Trop Dis. 2024 May 13;18(5):e0012136. doi: 10.1371/journal.pntd.0012136 (PMC11090343; doi:10.1371/journal.pntd.0012136)
Supplement: S5 Table — The fatality rates of active TB-COVID co-infection (n = 11). (PDF) [file pntd.0012136.s005.pdf]

S5 Table The fatality rates of active and previous TB-COVID coinfection (n=11)

First author (year)	Country	Study design	Single center	Time	Sample size	Age	Gender	Comorbidity	% BCG vaccination
Sy 2020	Philippines	retrospective observational study	no	May 17, 2020 to June 15, 2020	total: 106<br>in-hospital :66	total: 50.33(21.68)	48(72.7)	Hypertension 16(24.2)<br>Diabetes 10(15.2)<br>Cancer 1(1.5)<br>Renal cancer 4(6.1)<br>Cardiac disease 7(10.6)<br>Asthma 1(1.5)<br>COPD 1(1.5)	/
Stochino 2020	Italy	retrospective observational study	yes	/	20	39(27-47)	12 (60%)	/	3(15%)
Davies 2021	South Africa	retrospective	no	Till Mar.	total: 343	/	/	/	/

		observational study		1st 2020	in-hospital :148				
Hassan 2023	Pakistan	retrospective observational study	yes	February 2022 - August 2022	in-hospital :218	/	male: 170 (77.98%)	without-COPD	/
Liubov Parolina 2022	Russian	retrospective observational study	2	October 2020 to August 2021	in-hospital :75	45-52	male: 49 (65.3)	Cardiovascular disease 25 (33.3) Chronic respiratory disease 12 (16.0) Chronic liver disease 17 (22.7) Diabetes mellitus 8 (10.7) Chronic renal disease 5 (6.7)	/

								Chronic gastrointestinal tract disease 5 (6.7)<br>Nervous system diseases 13 (17.3)	
Wang 2022 (Omicron variant)	Changchun, China	retrospective observational study	yes	March 2022 to June 2022	129	/	/	/	/
Adzic-Vukicevic 2022	Serbia	retrospective observational study	/	6 March 2020 to 1 April 2022	53	/	male 35 (66)	Cardiac disease 12 (22.6)<br>Respiratory disease 19 (35.8)<br>Diabetes 9 (17.0)	/
Malashenkov 2021	St. Petersburg, Russia	retrospective observational study	yes	/	63	44.8 (24-86)	male 47 (74.6)	HIV 30 (53.5)	/

/: No information provided

S5 Table Continued The fatality rates of active TB-COVID coinfection (n=11)

First author (year)	% extrapulmonary TB	% drug resistance	% active TB	% has TB before COVID-19	% Symptomatic (yes or no)	% ICU	Treatment	Died	Fatality rate (%)
Sy 2020	/	/	100%	/	/	/	/	total: 25 in-hospital: 18	23.6 (total) 27.3 (in-hospital)
Stochino 2020	3(15%)	5(40%)	100%	/	100%	/	ATT, HQC	1	5 (in-hospital)
Davies 2021	/	/		/	/	/	/	total: 26 in-hospital: 25	7.6 (total) 16.9 (in-hospital)

Hassan 2023	/	/	100%	/	100%	/	/	51	23.39% (in hospital)
Liubov Parolina 2022	/	42(56%)	100%%	100%%	100%	/	/	7	9.3%(in hospital)
Wang 2022 (Omicron variant)	/	/	100%	/	100%	/	/	0	0 (in hospital)
Adzic-Vukicevic 2022	1 (2.0)	2 (3.8)	100%	/	1	/	Oxygen support via high-flow nasal cannula and later noninvasive ventilation; Antiviral drugs (favipiravir, molnupiravir, and remdesivir); Immunomodulatory	1	1.9% (in hospital)

							treatment; anticoagulation therapy; azithromycin		
Malashenkov 2021	23 (36.5)	/	100%	/	1	/	ATT	13 (10 HIV, 3 no HIV)	10% (in hospital, exclude HIV)
