# Supplementary material for: Systematic review and meta-analysis of Tuberculosis and COVID-19 Co-infection: Prevalence, fatality, and treatment considerations
Source: PLoS Negl Trop Dis. 2024 May 13;18(5):e0012136. doi: 10.1371/journal.pntd.0012136 (PMC11090343; doi:10.1371/journal.pntd.0012136)
Supplement: S6 Table — (PDF) [file pntd.0012136.s006.pdf]

S6 Table The fatality rates of previous TB-COVID coinfection (n=3)

First author (year)	Country	Study design	Single center	Time	Sample size	Died	Fatality rate (%)
Davies 2021	South Africa	retrospective observational study	no	Till Mar. 1st 2020	total: 1785<br>in-hospital :321	total: 87<br>in-hospital: 77	4.9 (total)<br>24.0 (in-hospital)
The GTN 2022	172 centres from 34 countries (Argentina, Belarus, Belgium, Brazil, Chile, China, France, Republic of Guinea, India, Italy, Mexico, Niger, Panama, Peru, Portugal, Romania, Russia, Singapore, Spain, Switzerland , UK， Australia, Canada, Colombia, Greece, Honduras, Lithuania, the Netherlands, Oman, Paraguay, Serbia, Slovakia, South Africa and USA.)	prospective cohort study	172	March 2020-June 2021	234	34	14.5 (total)

Wang 2022 (Omicron variant)	Changchun, China	retrospective observational study	yes	March 2022 to June 2022	24	0	0 (in hospital)
