# Supplementary material for: Systematic review and meta-analysis of Tuberculosis and COVID-19 Co-infection: Prevalence, fatality, and treatment considerations
Source: PLoS Negl Trop Dis. 2024 May 13;18(5):e0012136. doi: 10.1371/journal.pntd.0012136 (PMC11090343; doi:10.1371/journal.pntd.0012136)
Supplement: S7 Table — (PDF) [file pntd.0012136.s007.pdf]

S7 Table Quality Assessment of Each Included Study

Study	Q1	Q2	Q3	Q4	Q5	Q6	Q7	Q8	Q9
Gupta 2020	YES	YES	NO	YES	YES	YES	YES	YES	YES
Motta 2020	UNCLEAR	UNCLEAR	NO	YES	YES	YES	YES	YES	YES
Sy 2020	YES	YES	UNCLEAR	YES	YES	YES	YES	YES	NO
Stochino 2020	YES	YES	NO	YES	YES	YES	YES	YES	YES
Davies 2021	NO	YES	YES	YES	YES	YES	UNCLEAR	YES	YES
Domingo 2020	UNCLEAR	YES	NO	YES	YES	YES	YES	YES	YES
Gubkina	NO	YES	NO	YES	YES	YES	YES	YES	YES
Hassan 2023	NO	YES	UNCLEAR	YES	YES	YES	YES	YES	YES
Parolina 2022	UNCLEAR	UNCLEAR	NO	YES	YES	YES	YES	YES	YES
Sereda 2022	NO	NO	NO	YES	YES	YES	YES	YES	YES
Nabity 2021	NO	YES	YES	YES	YES	YES	UNCLEAR	YES	YES
The GTN 2022	UNCLEAR	UNCLEAR	YES	YES	YES	YES	YES	YES	YES
Wang 2022	UNCLEAR	YES	UNCLEAR	YES	YES	YES	YES	YES	YES
Adzic-Vukicevic 2022	YES	UNCLEAR	NO	NO	YES	YES	YES	YES	YES

Otlu 2022	UNCLEAR	UNCLEAR	NO	NO	NO	YES	YES	YES	YES
Siranart 2023	UNCLEAR	YES	NO	YES	YES	YES	YES	YES	NO
Malashenkov 2021	UNCLEAR	UNCLEAR	NO	YES	YES	YES	YES	YES	YES

Note: Four options for each question: yes, no, unclear and not appliable. Note: Four options for each question: yes, no, unclear and not appliable. Q1: Was the sample frame appropriate to address the target population? Q2: Were study participants sampled in an appropriate way? Q3: Was the sample size adequate? Q4: Were the study subjects and the setting described in detail? Q5: Was the data analysis conducted with sufficient coverage of the identified sample? Q6: Were valid methods used for the identification of the condition? Q7: Was the condition measured in a standard, reliable way for all participants? Q8: Was there appropriate statistical analysis? Q9: Was the response rate adequate, and if not, was the low response rate managed appropriately?
