# Supplementary material for: Systematic review and meta-analysis of Tuberculosis and COVID-19 Co-infection: Prevalence, fatality, and treatment considerations
Source: PLoS Negl Trop Dis. 2024 May 13;18(5):e0012136. doi: 10.1371/journal.pntd.0012136 (PMC11090343; doi:10.1371/journal.pntd.0012136)
Supplement: S8 Table — (PDF) [file pntd.0012136.s008.pdf]

S8 Table Egger's Test on Total Fatality Rate

<table>
<tr><th>Group</th><th>Result</th></tr>
<tr><td>All included studies, total fatality rate</td><td>Number of studies = 6   Root MSE = 7.307
<table>
<tr><th>Std_Eff</th><th>Coefficient</th><th>Std. err.</th><th>t</th><th>P>|t|</th><th>[95% conf.</th><th>interval]</th></tr>
<tr><td>slope</td><td>.077232</td><td>.0364415</td><td>2.12</td><td>0.101</td><td>-.0239458</td><td>.1784097</td></tr>
<tr><td>bias</td><td>-1.221472</td><td>5.712615</td><td>-0.21</td><td>0.841</td><td>-17.08223</td><td>14.63929</td></tr>
</table>
Test of H0: no small-study effects   P = 0.841</td></tr>
<tr><td>LMICs subgroup, total fatality rate</td><td>Number of studies = 5   Root MSE = 4.617
<table>
<tr><th>Std_Eff</th><th>Coefficient</th><th>Std. err.</th><th>t</th><th>P>|t|</th><th>[95% conf.</th><th>interval]</th></tr>
<tr><td>slope</td><td>.0212155</td><td>.0322595</td><td>0.66</td><td>0.558</td><td>-.0814486</td><td>.1238796</td></tr>
<tr><td>bias</td><td>2.790649</td><td>3.980939</td><td>0.70</td><td>0.534</td><td>-9.878474</td><td>15.45977</td></tr>
</table>
Test of H0: no small-study effects   P = 0.534</td></tr>
<tr><td>HICs subgroup, total fatality rate</td><td>N/A</td></tr>
</table>
