# Supplementary material for: Systematic review and meta-analysis of Tuberculosis and COVID-19 Co-infection: Prevalence, fatality, and treatment considerations
Source: PLoS Negl Trop Dis. 2024 May 13;18(5):e0012136. doi: 10.1371/journal.pntd.0012136 (PMC11090343; doi:10.1371/journal.pntd.0012136)
Supplement: S9 Table — (PDF) [file pntd.0012136.s009.pdf]

S9 Table Sensitives Analysis on MA of Total Fatality Rate

Group	Study omitted	Estimate	[95% Conf.	Interval]
All included studies, total fatality rate	Davies 2021	.08420974	.02916992	.13924957
	Sy 2020	.05693926	.01695497	.09692354
	Nabity 2021	.06916133	.02728125	.11104142
	Kayal 2022	.08809815	.0526202	.12357611
	The GTN 2022	.06832158	.02432335	.1123198
	Otlu 2022	.08799348	.04524193	.13074502
	Combined	.07545767	.03636894	.1145464
LMICs subgroup, total fatality rate	Davies 2021	.07242081	.01319041	.1316512
	Sy 2020	.03959082	.00565406	.07352759
	Kayal 2022	.07919252	.03585572	.12252931
	Otlu 2022	.07602475	.03132443	.12072506
	The GTN 2022	.05458668	.01252823	.09664512
	Combined	.06185261	.02428194	.09942327
HICs subgroup, total fatality rate	Nabity 2021	.14186851	.10164137	.18209565
	The GTN 2022	.10047771	.09304223	.10791319
	Combined	.11625825	.07685645	.15566004
