# Supplementary material for: Systematic review and meta-analysis of Tuberculosis and COVID-19 Co-infection: Prevalence, fatality, and treatment considerations
Source: PLoS Negl Trop Dis. 2024 May 13;18(5):e0012136. doi: 10.1371/journal.pntd.0012136 (PMC11090343; doi:10.1371/journal.pntd.0012136)
Supplement: S10 Table — (PDF) [file pntd.0012136.s010.pdf]

S10 Table Egger's Test on MA of In-Hospital Fatality Rate

Group	Result
All included studies, total fatality rate	Number of studies = 12 Root MSE = 3.449<br>Std_Eff | Coefficient | Std. err. | t | P>|t| | [95% conf. interval]<br>slope | -.0057572 | .0340665 | -0.17 | 0.869 | -.081662 | .0701476<br>bias | 3.56432 | 1.57698 | 2.26 | 0.047 | .0505893 | 7.07805<br>Test of H0: no small-study effects P = 0.047
LMICs subgroup, total fatality rate	Number of studies = 8 Root MSE = 4.193<br>Std_Eff | Coefficient | Std. err. | t | P>|t| | [95% conf. interval]<br>slope | -.0223331 | .0460047 | -0.49 | 0.645 | -.1349025 | .0902363<br>bias | 4.945422 | 2.524951 | 1.96 | 0.098 | -1.232911 | 11.12376<br>Test of H0: no small-study effects P = 0.098
HICs subgroup, total fatality rate	Number of studies = 4 Root MSE = .7164<br>Std_Eff | Coefficient | Std. err. | t | P>|t| | [95% conf. interval]<br>slope | .0235276 | .051224 | 0.46 | 0.691 | -.1968714 | .2439266<br>bias | 1.723232 | 1.029584 | 1.67 | 0.236 | -2.706709 | 6.153174<br>Test of H0: no small-study effects P = 0.236
