# Supplementary material for: Systematic review and meta-analysis of Tuberculosis and COVID-19 Co-infection: Prevalence, fatality, and treatment considerations
Source: PLoS Negl Trop Dis. 2024 May 13;18(5):e0012136. doi: 10.1371/journal.pntd.0012136 (PMC11090343; doi:10.1371/journal.pntd.0012136)
Supplement: S11 Table — (PDF) [file pntd.0012136.s011.pdf]

S11 Table Sensitives Analysis on MA of In-Hospital Fatality Rate

Group	Result
All included studies, total fatality rate	see below

```
----------------------------------------------------------------------------
Study omitted      |   Estimate       [95%  Conf.  Interval]
-------------------+--------------------------------------------------------
Wang 2022          |   .13987198      .07331558    .20642837
Sy 2020            |   .113248        .0500706     .17642541
Stochino 2020      |   .11925589      .05540966    .18310213
Siranart 2023      |   .13073454      .06470376    .19676533
Sereda 2022        |   .13867915      .06788358    .20947471
Parolina 2022      |   .13002032      .06259271    .19744793
Malashenkov 2021   |   .12862618      .06281467    .19443769
Hassan 2023        |   .11332622      .05234266    .17430978
Gupta 2020         |   .11780328      .0541173     .18148926
Domingo 2020       |   .12960816      .06392144    .19529488
Davies 2021        |   .11176513      .05660023    .16693002
Adzic-Vukicevic 2022|  .13930587      .06712471    .21148704
-------------------+--------------------------------------------------------
Combined           |   .12617177      .06374906    .18859448
----------------------------------------------------------------------------
```

LMICs subgroup, total fatality rate	see table below

Study omitted	Estimate	[95% Conf.	Interval]
Wang 2022	.15062528	.06043781	.24081275
Sy 2020	.11055006	.02961115	.19148898
Siranart 2023	.13645513	.05139911	.22151115
Sereda 2022	.1480833	.05478438	.24138222
Hassan 2023	.111431	.03325019	.18961181
Gupta 2020	.11682395	.0355487	.1980992
Davies 2021	.11008606	.04029906	.17987307
Adzic-Vukicevic 2022	.1489662	.05313799	.24479442
Combined	.1292741	.0506797	.20786851

HICs subgroup, total fatality rate

Study omitted	Estimate	[95% Conf.	Interval]
Stochino 2020	.09358173	.0431321	.14403137
Parolina 2022	.11873809	.04081912	.19665706
Malashenkov 2021	.10948133	.04276124	.17620142
Domingo 2020	.11147919	.04860135	.17435703
Combined	.10390648	.0551503	.15266267

.
