# Supplementary material for: Systematic review and meta-analysis of Tuberculosis and COVID-19 Co-infection: Prevalence, fatality, and treatment considerations
Source: PLoS Negl Trop Dis. 2024 May 13;18(5):e0012136. doi: 10.1371/journal.pntd.0012136 (PMC11090343; doi:10.1371/journal.pntd.0012136)
Supplement: S12 Table — (PDF) [file pntd.0012136.s012.pdf]

S12 Table Egger's Test on MA of active/previous TB-COVID Coinfection Fatality Rate

Group	Result
Total active TB-COVID coinfection patients	N/A
Total previous TB-COVID coinfection patients	N/A
Hospitalized active TB-COVID coinfection patients	Number of studies = 8 Root MSE = 2.694<br>Std_Eff | Coefficient Std. err. t P>|t| [95% conf. interval]<br>slope | -.0183222 .0183872 -1.00 0.357 -.0633141 .0266698<br>bias | 4.140104 1.308575 3.16 0.019 .9381356 7.342072<br>Test of H0: no small-study effects P = 0.019
Hospitalized previous TB-COVID coinfection patients	N/A
