# Supplementary material for: Systematic review and meta-analysis of Tuberculosis and COVID-19 Co-infection: Prevalence, fatality, and treatment considerations
Source: PLoS Negl Trop Dis. 2024 May 13;18(5):e0012136. doi: 10.1371/journal.pntd.0012136 (PMC11090343; doi:10.1371/journal.pntd.0012136)
Supplement: S13 Table — (PDF) [file pntd.0012136.s013.pdf]

S13 Table Sensitives Analysis on MA of In-Hospital Fatality Rate

<table>
<tr><th>Group</th><th>Result</th></tr>
<tr><td>Total active TB-COVID coinfection patients</td><td>N/A</td></tr>
<tr><td>Total previous TB-COVID coinfection patients</td><td>N/A</td></tr>
<tr><td>Hospitalized active TB-COVID coinfection patients</td><td>
<table>
<tr><th>Study omitted</th><th>Estimate</th><th>[95% Conf.</th><th>Interval]</th></tr>
<tr><td>Davies 2021</td><td>.1048543</td><td>.03325595</td><td>.17645264</td></tr>
<tr><td>Sy 2020</td><td>.09419251</td><td>.02598333</td><td>.1624017</td></tr>
<tr><td>Stochino 2020</td><td>.12193917</td><td>.04640626</td><td>.19747209</td></tr>
<tr><td>Parolina 2022</td><td>.11684211</td><td>.03979505</td><td>.19388917</td></tr>
<tr><td>Malashenkov 2021</td><td>.11529269</td><td>.04094747</td><td>.1896379</td></tr>
<tr><td>Adzic-Vukicevic 2022</td><td>.12987042</td><td>.03773961</td><td>.22200123</td></tr>
<tr><td>Hassan 2023</td><td>.09191681</td><td>.03268274</td><td>.15115088</td></tr>
<tr><td>Wang 2022</td><td>.1320854</td><td>.05478874</td><td>.20938205</td></tr>
<tr><td>Combined</td><td>.11351642</td><td>.04437119</td><td>.18266166</td></tr>
</table>
</td></tr>
<tr><td>Hospitalized previous TB-COVID coinfection patients</td><td>N/A</td></tr>
</table>
