# Supplementary material for: Systematic review and meta-analysis of Tuberculosis and COVID-19 Co-infection: Prevalence, fatality, and treatment considerations
Source: PLoS Negl Trop Dis. 2024 May 13;18(5):e0012136. doi: 10.1371/journal.pntd.0012136 (PMC11090343; doi:10.1371/journal.pntd.0012136)
Supplement: S14 Table — (PDF) [file pntd.0012136.s014.pdf]

S14 Table Sensitives Analysis on RR of in-hospital fatality between TB-COVID patients and single COVID patients

Group	Result
in-hospital fatality between TB-COVID patients and single COVID patients	

Study omitted	Estimate	[95% Conf.	Interval]
Parolina 2022	1.8891293	1.137914	3.1362734
Sereda 2022	.76078881	.10897186	5.3114595
Sy 2020	.36048472	.13458119	.96558247
Combined	.80498853	.17622672	3.6771184
