# Supplementary figures and images for: Systematic review and meta-analysis of Tuberculosis and COVID-19 Co-infection: Prevalence, fatality, and treatment considerations
Source: PLoS Negl Trop Dis. 2024 May 13;18(5):e0012136. doi: 10.1371/journal.pntd.0012136 (PMC11090343; doi:10.1371/journal.pntd.0012136)

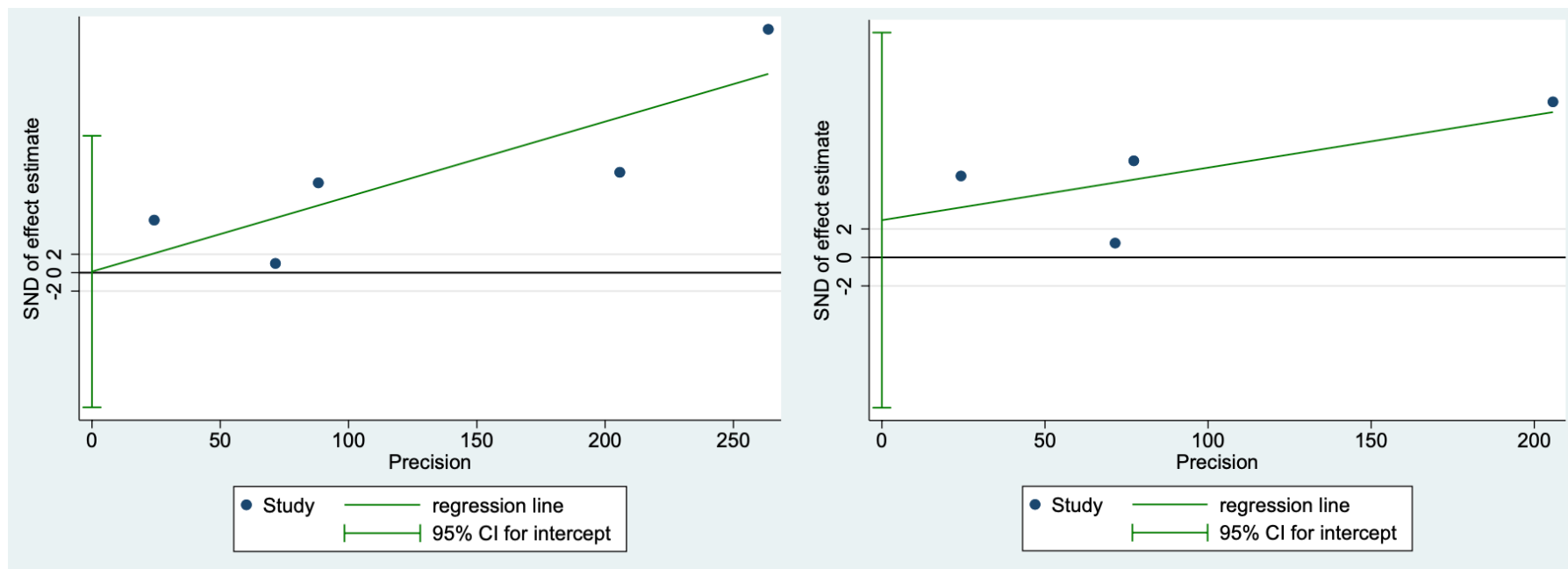

S2 Fig Egger's Test on MA of Total Fatality Rate

Supplement: S2 Fig — (PDF) [file pntd.0012136.s016.pdf]

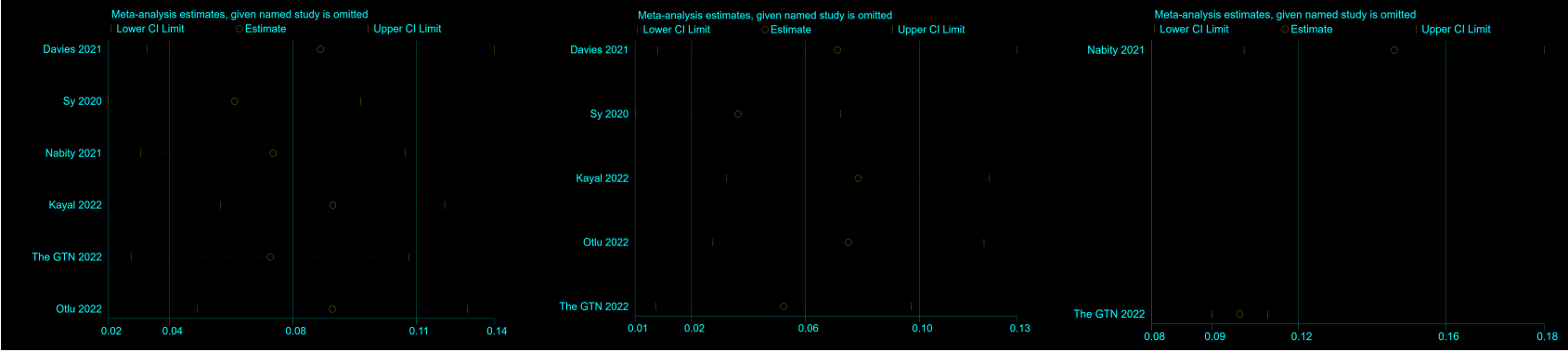

S3 Fig Sensitives Analysis on MA of Total Fatality Rate

Supplement: S3 Fig — (PDF) [file pntd.0012136.s017.pdf]

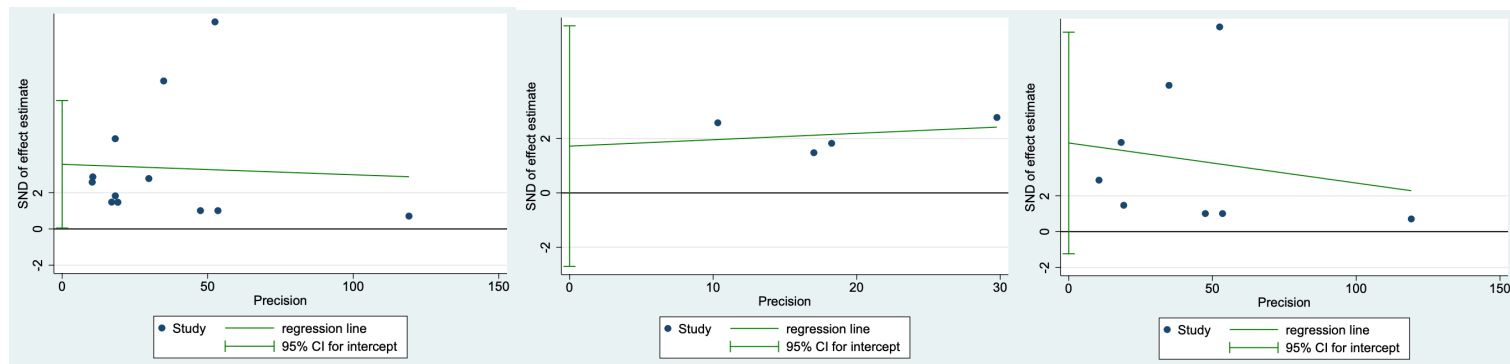

S4 Fig Egger's Test on MA of In-Hospital Fatality Rate

Supplement: S4 Fig — (PDF) [file pntd.0012136.s018.pdf]

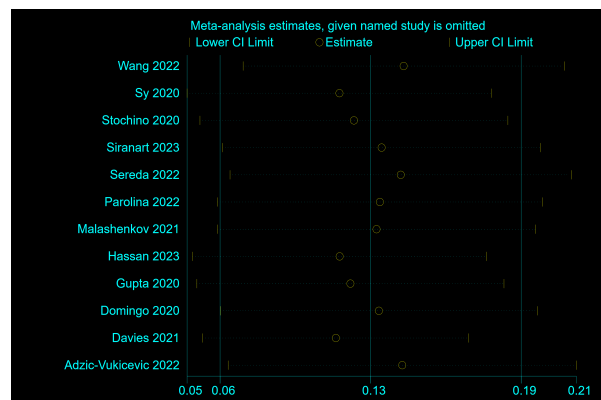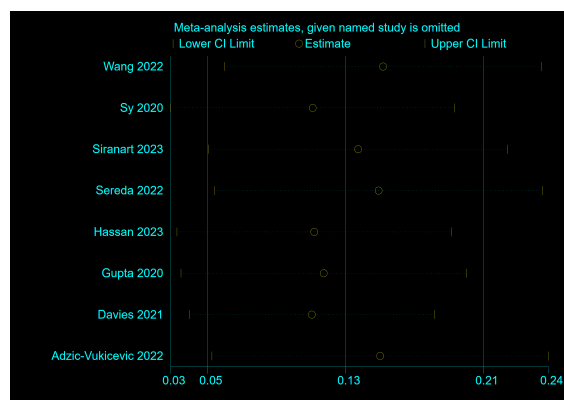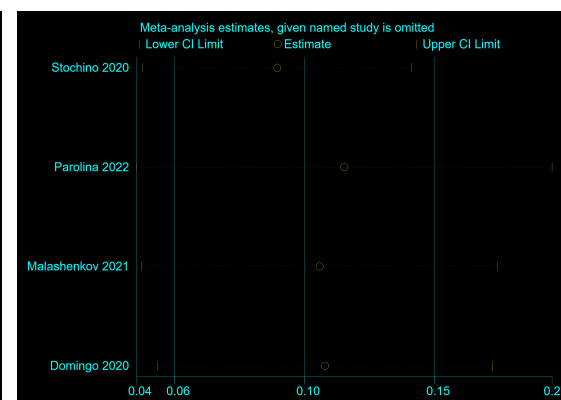

S5 Fig Sensitives Analysis on MA of In-Hospital Fatality Rate

Supplement: S5 Fig — (PDF) [file pntd.0012136.s019.pdf]

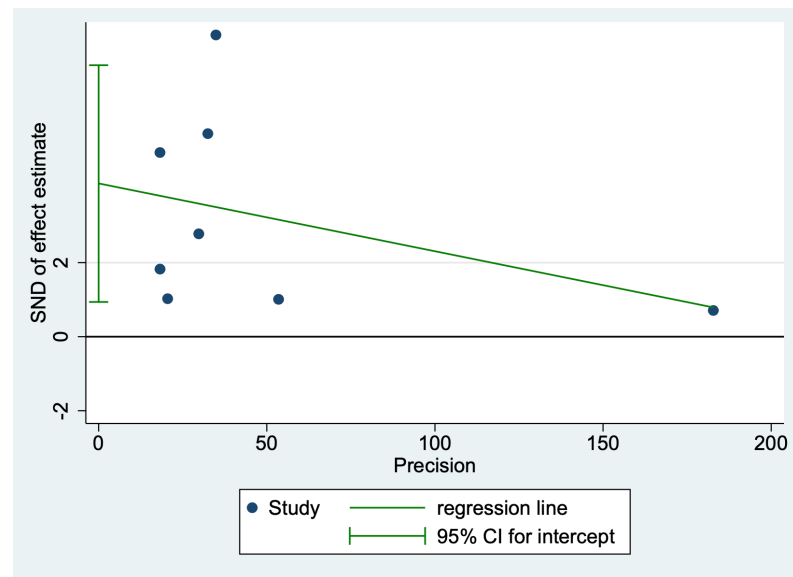

S6 Fig Egger's Test on MA of Hospitalized Active TB-COVID Coinfection Patients Fatality Rate

Supplement: S6 Fig — (PDF) [file pntd.0012136.s020.pdf]
