# Supplementary material for: Systematic review and meta-analysis of Tuberculosis and COVID-19 Co-infection: Prevalence, fatality, and treatment considerations
Source: PLoS Negl Trop Dis. 2024 May 13;18(5):e0012136. doi: 10.1371/journal.pntd.0012136 (PMC11090343; doi:10.1371/journal.pntd.0012136)
Supplement: S8 Fig — (PDF) [file pntd.0012136.s022.pdf]

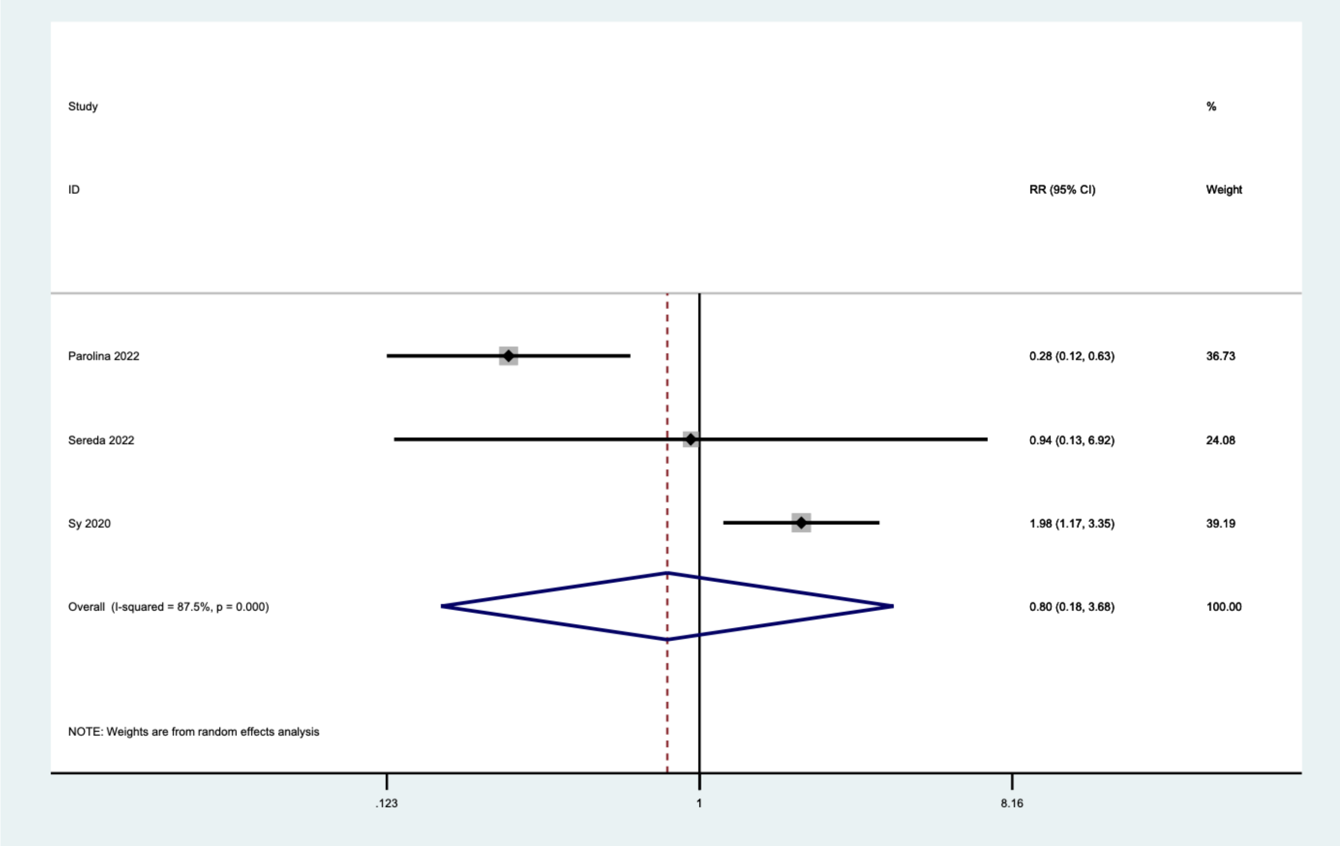

S8 Fig Relative Risk of in-hospital Fatality between TB-COVID Coinfection and Single COVID-19 Coinfection
