# Supplementary material for: Systematic review and meta-analysis of Tuberculosis and COVID-19 Co-infection: Prevalence, fatality, and treatment considerations
Source: PLoS Negl Trop Dis. 2024 May 13;18(5):e0012136. doi: 10.1371/journal.pntd.0012136 (PMC11090343; doi:10.1371/journal.pntd.0012136)
Supplement: S10 Fig — (PDF) [file pntd.0012136.s024.pdf]

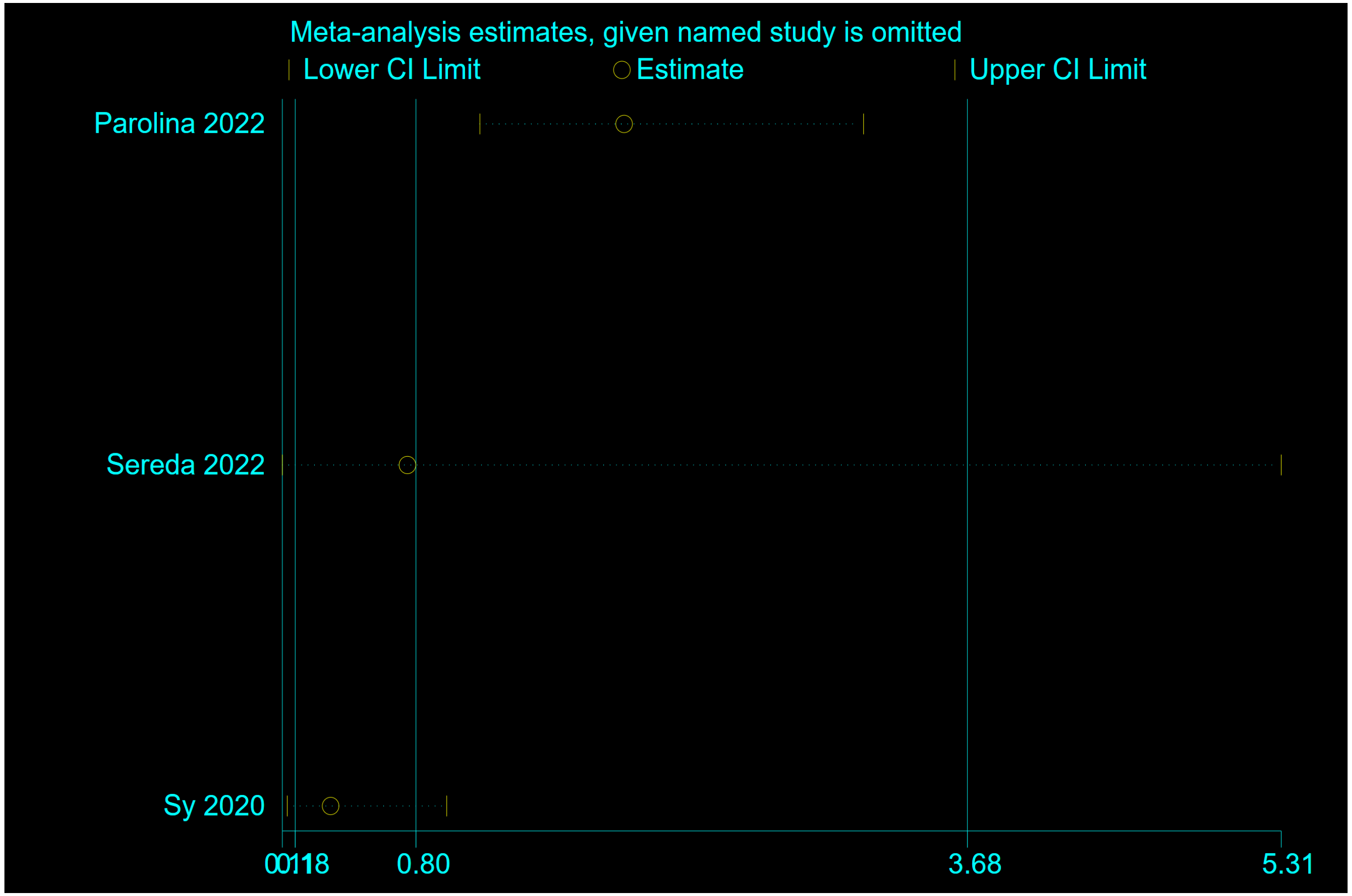

S10 Fig Sensitives Analysis on RR of in-hospital fatality between TB-COVID patients and single COVID patients
